# HDAC4/MybL1/YAP novel signaling axis is required for pancreatic cancer metastasis to the liver

**DOI:** 10.7150/ijbs.102132

**Published:** 2025-10-24

**Authors:** Mouad Edderkaoui, Omer H.M. Elmadbouh, Adrian Lim, Yan Ou, Dina Hauptschein, Ankita Guha, Abdo Darwish, Vinicius F. Calsavara, Ramachandran Murali, Neil Bhowmick, Arsen Osipov, Angela J. Mathison, Raul Urrutia, Qiang Wang, Stephen J. Pandol

**Affiliations:** 1 Departments of Medicine and Biomedical Sciences, Samuel Oschin Comprehensive Cancer Center, Cedars-Sinai Medical Center (CSMC), Los Angeles, California, USA.; 2 Department of Biomedical Sciences, Research Division of Immunology, CSMC, USA.; 3 Department of Medicine, UCLA, California, USA.; 4 Linda T. and John A. Mellowes Center for Genomic Sciences and Precision Medicine, Medical College of Wisconsin, 8701 Watertown Plank Road, Milwaukee, WI, 53226, USA.; 5 Department of Surgery, Medical College of Wisconsin, 8701 Watertown Plank Road, Milwaukee, WI, 53226, USA.; 6 Department of Biochemistry, Medical College of Wisconsin, 8701 Watertown Plank Road, Milwaukee, WI, 53226, USA.; 7 David Geffen School of Medicine at UCLA, Los Angeles, CA, USA.; 8 Department of Internal Medicine, Creighton University Health Education Alliance, Phoenix, AZ, USA.; 9 Cedars-Sinai Medical Center, Biostatistics Shared Resource, Department of Computational Biomedicine, Los Angeles, California, USA.

## Abstract

Pancreatic ductal adenocarcinoma (PDAC) is one of the deadliest forms of human malignancy, and there is an urgency to develop more effective therapy. We previously showed that Metavert, a dual inhibitor of glycogen synthase kinase 3-beta (GSK-3β) and histone deacetylases (HDACs) prevents pancreatic ductal adenocarcinoma (PDAC) metastasis. In this study, we investigated the mechanisms that mediate metastasis and the roles of GSK-3β, HDACs, and Yes-associated protein (YAP) in this process.

We found that HDAC4 and YAP are highly expressed in PDAC from patients with rapid disease progression and metastasis compared to those with prolonged recurrence-free survival. Pan-HDAC inhibition decreases metastasis in the splenic PDAC metastatic mouse model. Inhibition of HDAC4 reduces migration of cancer cells and decreases the mRNA and protein levels of transcription factor MYB Proto-Oncogene Like 1 (MybL1) and YAP. Mechanistic studies show that HDAC4 regulates transcription of YAP through up-regulating MybL1 expression. Comparable results were observed in colon and prostate cancers. ATAC-seq studies show that inhibition of HDAC4 causes chromatin remodeling in the YAP promoter region and reduces accessibility to the binding sites of multiple transcription factors, including those of MybL1. Pharmacological or molecular inhibition of YAP significantly decreases PDAC metastasis in vivo. Imaging Mass Cytometry (IMC) reveals no significant changes in immune cells, but a notable shift in the distribution patterns of cancer-associated hepatic stellate cells in the metastatic niche, when YAP is ablated in the cancer cells.

The results demonstrate a novel metastasis-driving cell signaling pathway mediated by the functional interaction between HDAC4 and MybL1, which regulates YAP expression and metastasis.

## Introduction

Pancreatic ductal adenocarcinoma (PDAC) is a disease with minimally effective treatments and with the lowest survival rates of any cancer^1^. One of the primary reasons for such a poor outcome is the rapid metastatic rate of PDAC and activation of multiple pro-cancer pathways in the cancer cells, which allow PDAC cells to overcome treatments based on the inhibition of a single oncogenic pathway.

Recently, we have shown that simultaneous targeting of Glycogen Synthase Kinase-3 Beta (GSK-3β) and HDAC by the dual inhibitor Metavert significantly increased the survival of mice with PDAC and completely prevented metastasis in the transgenic LSL-*Kras^G12D^*^/+^; LSL-Trp53^R172H/+^; Pdx-1-Cre (KPC) mice and a syngeneic mouse model of pancreatic cancer^2^. The results suggested that GSK-3β and/or HDAC could be essential mediators of PDAC metastasis. Metavert inhibits all members of the HDAC classes I and II. Therefore, we investigated which HDAC is involved in mediating metastasis. We uncovered that HDAC4 is the most critical HDAC for regulating metastasis in PDAC. The protein level HDAC4 is increased in PDAC tumors with metastasis compared to those without metastasis. The transcription factor Sp1 can mediate the regulation of HDAC4^3^, the expression of which is associated with increased metastasis in PDAC patients, compared to absence of Sp1 in patients with no metastasis^4^.

YAP is a transcriptional coactivator that exhibits oncogenic activities and is upregulated in most solid tumors, including pancreatic cancer^5^-^16^. YAP is vital in regulating proliferation, tumor progression, and drug resistance^5^, ^17^, ^18^. When activated, YAP translocates into the nucleus and mediates gene transcription by binding to transcription factors, such as the TEA domain family (TEAD) proteins^19^. Phosphorylation/inhibition of YAP has been shown to reduce metastasis in pancreatic cancer^20^.

Very little is known about the interaction between HDAC4 and YAP and how they work together to regulate cancer promotion. Nothing is known about HDAC4 regulating the transcription or translation of YAP. In this study, we investigated the mechanism through which HDAC4 and YAP regulate metastasis in PDAC. A recent study showed that YAP promotes epithelial to mesenchymal transition (EMT) and invasion through transcriptional activation of the Rho nucleotide guanosine triphosphate (Rho GTPase) activating protein 29, which reduces cytoskeletal rigidity and promotes a metastatic phenotype^21^. However, there is no data on the direct effect of YAP on metastasis in a metastatic animal model.

Our studies demonstrate a hitherto unknown interaction between HDAC4 and YAP responsible for regulating metastasis in cancers, including PDAC. We found that HDAC4 regulates transcription of YAP through mediating the interdependent regulation between transcription factors MybL1 and YAP. Our data indicate that HDAC4 up-regulates MybL1 expression, which then transcriptionally regulates YAP expression. We also found a positive feedback loop regulating the expression of MybL1 and YAP. Inhibition of the HDAC4/MybL1/YAP signaling pathway decreased EMT, migration, and metastasis.

The novel cell-signaling pathway identified here will allow us to understand the mechanism of metastasis better and therefore, develop therapeutics to limit cancer metastasis.

## Material and Methods

**Chemicals:** Metavert was synthesized by Royal Pharma (Mumbai, India). Saha was from Cayman (Ann Arbor, MI). Tideglusib and TH34 were from MedChemExpress (Monmouth Junction, NJ). Lmk235 was from Medkoo Biosciences (Morrisville, NC), and Verteporfin was from AdooQ (Irvine, CA). All other chemicals were from Sigma Aldrich. HDAC4, HDAC10, and YAP antibodies were purchased from AbCam (Cambridge, MA). HDAC10 and MybL1 antibodies were purchased from Sigma-Aldrich (St. Louis, MO). YAP and CK19 antibodies (for IMC studies) were from Proteintech (Rosemont, IL). All other antibodies were obtained from Cell Signaling (Danvers, MA).

**Cell culture experiments:** The poorly differentiated MIA PaCa-2 and PANC-1, and moderately differentiated BxPC-3 human pancreatic ductal adenocarcinoma (PDAC) cell lines were obtained from the American Type Culture Collection (ATCC) in Manassas, VA. MIA PaCa-2 cells were grown in 1:1 D-MEM/F-12 medium supplemented with 10% fetal bovine serum (FBS), 4 mM l-glutamine, and 1% antibiotic/antimycotic solution. BxP-C3 cells were grown in RPMI-1640 medium supplemented with 10% fetal bovine serum (FBS) and 1% antibiotic/antimycotic solution. Cells were maintained at 37 ºC in a humidified atmosphere containing 5% CO_2_ and were used between passages 2 and 10.

Cells were transfected using Lipofectamine (Thermo Fisher, Canoga Park, CA) according to the company's protocol. YAP siRNAs (D-012200-01-0005 and D-012200-02-0005) and MybL1 siRNAs (D-010526-02-0002 and D-010526-01-0002) were from Horizon Discovery (Boyertown, PA). HDAC4 siRNA was from Cell Signaling. HDAC10 siRNA was from Sigma-Aldrich. And YAP and MybL1 plasmids were from Addgene (Watertown, MA).

**Human samples:** PDAC and liver tissues from patients with and without metastasis were provided to us by the University of Nebraska Medical School through the Nebraska Rapid Autopsy Program. The Institutional Review Board (IRB) of Cedars-Sinai Medical Center (CSMC) approved the study protocol with the IRB protocol number 50715. CSMC provided PDAC tissues from patients with different days to recurrence of the disease through the approved IRB protocol numbers 1517 and 54363.

**Animals:** All animal studies were performed according to the guidance of the Institutional nimal Care and Use Committee (IACUC) and after the approval of the IACUC protocol # 8820 at the CSMC. All mice were housed in Association for Assessment and Accreditation of Laboratory Animal Care (AAALAC)- accredited facilities and used in accordance with the National Institutes of Health Guide for the Care and Use of Laboratory Animals.

**Figure 1:**
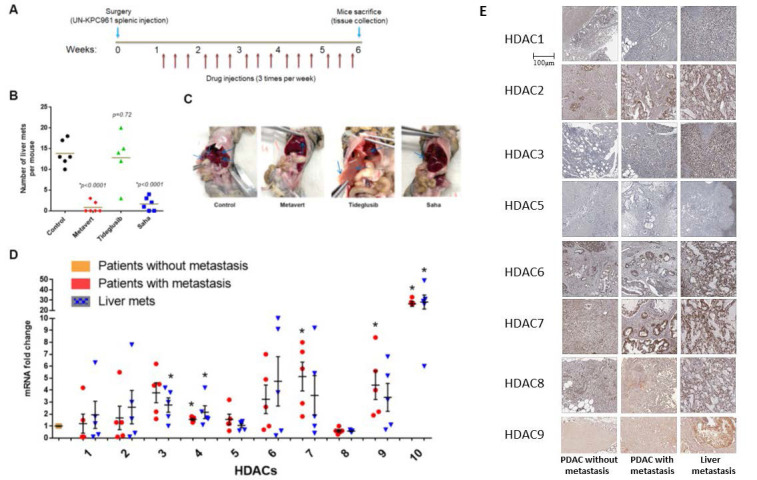
** HDACs, but not GSK-3β, are involved in mediating PDAC metastasis.** Mice were intraperitoneally (i.p) injected with GSK-3β inhibitor Tideglusib (50 mg/kg), HDAC pan-inhibitor Saha (50mg/kg), or dual inhibitor for GSK-3β and HDAC Metavert (10 mg/kg) 3 times/ week for 6 weeks (A). Number of metastatic lesions quantified in the liver (B) and images of representative mice shown (C). RT-PCR of HDACs from the pancreas and the liver of PDAC patients (N=5) (D). IHC of HDACs staining in human PDAC and liver metastasis tissues (E). *, *p* < 0.05 *versus* patients without metastasis.

1x10^6^ UNKPC961-Luc cells were injected into the spleen of B6.129J (2-3 months old males and females), followed by a treatment for 6 weeks with intraperitoneal (i.p.) injections (N = 6). In the first study, mice were injected with GSK-3β inhibitor Tideglusib (50 mg/Kg), HDAC pan-inhibitor Saha (50 mg/Kg), dual inhibitor for GSK-3β and HDAC Metavert (10 mg/Kg), or a vehicle 3 times/ week. In the second experiment, mice were injected intraperitoneally (i.p.) with YAP inhibitor Verteporfin (25 mg/kg) or saline twice weekly. In the third experiment, B6.129J mice were injected with 1 × 10^6 UNKPC961-Luc or UNKPC961-Luc/YAP KO cells into the spleen, followed by no treatment for 6 weeks (n = 5). Mice were then sacrificed, metastatic lesions quantified, and blood and organs collected for analysis by blinded personnel. The total number of animals used is 46. One mouse was excluded after being injured and died from an injection (tideglusib group, **Fig. 1**).

**Tissue immunostaining:** Human and mouse tissues were fixed with formalin and embedded in paraffin. Staining was performed as shown before^2^, ^22^. Human tissue samples were received from the University of Nebraska through the Rapid Autopsy program or from the Cedars-Sinai Pathology Core. They were used at the CSMC with IRB protocol number 50715. Image deconvolution and cytometry were performed using the Halo software to create deconvoluted images. The brightfield algorithm used for color deconvolution to separate chromogenic stains for analysis and make the fluorescent image is Indica Labs - Deconvolution v1.1.8.

**Western blot:** Cells were re-suspended in RIPA phosphorylation buffer (50 mM NaCl, 50 mM Tris/HCl pH 7.2, 1% deoxycholic acid, 1% Triton X-100, 0.1% SDS, 10 mM Na2HPO4 + NaH2PO4, 100 mM NaF, 2 mM Na_3_VO_4_, 80 μM glycerophosphate, 20% glycerol, 1 mM PMSF, 5 μg/ml each of pepstatin, leupeptin, chymostatin, antipain, and aprotinin). Lysates were then centrifuged for 15 minutes at 16,000 x g at 4 °C. SDS-PAGE was used to separate proteins in the supernatant, and they were then electrophoretically transferred to nitrocellulose or PVDF membranes. Non-specific binding was blocked for one hour with 5% bovine serum albumin or non-fat dry milk in Tris-buffered saline (4 mM Tris base, 100 mM NaCl, pH 7.5) containing 0.05% Tween 20. Membranes were incubated with primary antibody overnight at 4°C and then with peroxidase-conjugated secondary antibody for one hour. Blots were developed using SuperSignal Chemiluminescent Substrate (Pierce, Rockford, IL, USA).

**Real-time quantitative PCR (RT-PCR):** Total RNA was extracted using Trizol (Thermo Fisher, Canoga Park, CA). The reverse transcription reaction was performed using High-Capacity Reverse Transcription Kit (Thermo Fisher, Canoga Park, CA). RT-PCR was used to quantify mRNA levels using the iTaq Universal SYBR Green Supermix (Bio-Rad, Hercules, CA) and the Bio-Rad CFX96 platform, according to the manufacturer's protocol. Gene expression levels were normalized to that of GAPDH*.* Primers were purchased from Integrated DNA Technologies (IDT), San Diego, CA. The sequences of human primers used for RT-PCR were as follow: h-HDAC1-F: CCAAGTACCACAGCGATGAC, h-HDAC1-R: TGGACAGTCCTCACCAACG, h-HDAC2-F: TGAAGGAGAAGGAGGTCGAA, h-HDAC2-R: GGATTTATCTTCTTCCTTAACGTCTG, h-HDAC3-F: CACCATGCCAAGAAGTTTGA, h-HDAC3-R: CCCGAGGGTGGTACTTGAG, h-HDAC4-F: GGCCCACCGGAATCTGAAC, h-HDAC4-R: GAACTCTGGTCAAGGGAACTG, h-HDAC5-F: TCTTGTCGAAGTCAAAGGAGC, h-HDAC5-R: GAGGGGAACTCTGGTCCAAAG, h-HDAC6-F: AAGAAGACCTAATCGTGGGACT, h-HDAC6-R: GCTGTGAACCAACATCAGCTC, h-HDAC7-F: GGCGGCCCTAGAAAGAACAG, h-HDAC7-R: CTTGGGCTTATAGCGCAGCTT, h-HDAC8-F: TCGCTGGTCCCGGTTTATATC, h-HDAC8-R: TACTGGCCCGTTTGGGGAT, h-HDAC9-F: AGTAGAGAGGCATCGCAGAGA, h-HDAC9-R: GGAGTGTCTTTCGTTGCTGAT, h-HDAC10-F: CAGTTCGACGCCATCTACTTC, h-HDAC10-R: CAAGCCCATTTTGCACAGCTC, h-YAP-F: TAGCCCTGCGTAGCCAGTTA, h-YAP-R: TCATGCTTAGTCCACTGTCTGT, h-MybL1-F: AGGCAAGCAGTGTAGAGAAAGA, h-MybL1-R: CGATTTCCCAACCGCTTATGT, h-Snail1-F: TGCCCTCAAGATGCACATCCGA, h-Snail1-R: GGGACAGGAGAAGGGCTTCTC, h-Vimentin-F: TGTCCAAATCGATGTGGATGTTTC, h-Vimentin-R: TTGTACCATTCTTCTGCCTCCTG, h-Zeb1-F: GGCATACACCTACTCAACTACGG, h-Zeb1-R: TGGGCGGTGTAGAATCAGAGTC, h-GAPDH-F: CCAGGTGGTCTCCTCTGACTTCAACA, and h-GAPDH-R; AGGGTCTCTCTCTTCCTCTTGTGCTC.

**EMT and migration measurements:** EMT was assessed by measuring the levels of EMT markers using RT-PCR. Migration was evaluated using the Culture-Insert 2 Well in the Dish 35 mm Kit from ibidi (Martinsried, Germany) according to the manufacturer's instructions. Photographic images were acquired at various times using an inverted microscope, and the area occupied by migration was measured using ImageJ software.

**Chromatin immunoprecipitation (ChIP) Pull-down Assay:** ChIP Pull-down assay was performed by expressing the MybL1-V5 (DNASU, Tempe, AZ ) and the F-YAP (Addgene, Watertown, MA ) tagged proteins and running PCR for the promoter of YAP and MybL1 using the following primers: YAP-ChIP promoter-F: AGGGCGAGCGGGTCACGT, YAP-ChIP promoter-R: CGCCTCCTCTCGGCTCTT, MybL1-ChIP promoter-F: AGGAAGGGGAAATTCCATTAAA, and MybL1-ChIP promoter-R: CCCAGAAATCAACCATCCTCTA (IDT, San Diego, CA) following immunoprecipitation of the MyBL1-V5 and F-YAP proteins.

**Imaging Mass Cytometry (IMC):** IMC was performed using the Mass Cytometry Core in Cedars-Sinai, and analysis was performed using the HALO software.

**RNA sequencing (RNAseq):** Extracted RNA from MIA PaCa-2 and PANC-1 cells was quantified by fluorometric methods, and integrity was assessed with Fragment Analysis (Agilent). RNA libraries were prepared according to the manufacturer's protocols utilizing the NEBNext Poly(A) mRNA Magnetic Isolation Module (New England Biolab Inc., Ipswich, MA) and the IDT xGEN Broad-Range RNA library kit with unique dual indexing (Integrated DNA Technologies, Coralville, IA). Next Generation Sequencing was completed at the CSMC Applied Genomics, Computation, and Translational (AGCT) Core on the Illumina NovaSeq 6000 (Illumina, San Diego, CA). A single-end 75-base pair read generated ~35 million reads per sample. Bioinformatics analysis of the RNAseq data was performed at the Mellowes Center for Genomic Science and Precision Medicine (RRID: SCR_022926) at the Medical College of Wisconsin (MCW). Sequencing reads were aligned to the reference transcriptome (Gencode v32, based on Ensembl v98) and processed through the MAPR-Seq Workflow^25^, with differential expression analysis completed using Bioconductor and edgeR v3.8.6 software. Genes with false discovery rate (FDR) less than 5% and a log2 fold change ≥ 0.75 and ≤ -0.75 will initially be filtered and considered significantly differentially expressed. RITAN pathway overrepresentation^23^ with HALLMARK features provided insight into the biological pathways and processes essential for Verteporfin treatment.

**Assay for Transposase-Accessible Chromatin with sequencing (ATAC-seq):** MIA PaCa-2 cells treated with and without Lmk-235 (5µM) and Saha (5µM) for 48 hours were used in the ATAC-seq study. Libraries were prepared following standard protocols and sequenced using paired-end reads on an Illumina platform in the genomic core at the Cedars-Sinai Medical Center in Los Angeles, CA. Reads were aligned to the human reference genome (hg38) using Bowtie2, and open chromatin regions were identified using MACS2. Peak reproducibility across biological replicates was assessed using IDR thresholding. Chromatin accessibility at the MYBL1 and YAP loci, particularly in their promoter regions, was visualized using the Integrative Genomics Viewer (IGV). To identify potential transcription factor binding sites, peak sequences were extracted and subjected to motif discovery analysis using MEME.

**Statistics:** Statistical analyses were performed using Student's t-test, one-way ANOVA, or Fisher's exact test, as determined by GraphPad Prism (GraphPad Software). The log-rank (Mantel-Cox) test was used to analyze survival data. A *p-value* of less than 0.05 was considered statistically significant.

For animal studies, the central hypothesis to be tested is whether there is a difference in the number of metastatic lesions between the treatment groups and the vehicle control. Based on the preliminary data, we assumed an event rate of 13 lesions in the control group. For a sample size of 6 mice per group, we achieve 80% power to detect a difference of 6.08 between groups (treatment group versus control group) using a test for the difference between two Poisson rates at a 1.25% (α = 0.05/4) significance level, adjusted by the Bonferroni correction for 4 comparisons.

## Results

### HDACs, but not GSK-3β, are involved in mediating PDAC metastasis

Previously, we showed that dual inhibition of GSK-3β and HDACs prevents metastasis in KPC mice and in syngeneic mice with PDAC cells injected in the pancreas^2^. The KPC mice carry the K-*Ras* and p53 mutations and mimic the human PDAC disease in general^24^. To determine the role of GSK-3β and HDACs in mediating PDAC metastasis to the liver, we developed a metastatic model of PDAC by injecting UNKPC961-Luc cells in the spleen of syngeneic B6.129J mice^25^. After one week, the animals were then treated with the dual inhibitor Metavert (10mg/Kg), the GSK-3β inhibitor Tideglusib (50 mg/kg) or the pan-HDAC inhibitor Saha (50 mg/kg) for 6 weeks (**Fig. 1A**). We found that Metavert and Saha significantly decreased the number of liver metastatic lesions, compared to mice treated with vehicle, from an average of 14 to 1 and 2 liver lesions, respectively (**Fig. 1B**). Tideglusib alone did not have any significant effect on the number of lesions, compared to the control group (**Fig. 1B**). Four of the six mice treated with Metavert and two of the six mice treated with Saha did not develop any metastatic lesions, whereas all mice developed lesions in the control and the Tideglusib groups (**Fig. 1B**). The spreading of PDAC cells to the liver and to other organs such as the pancreas, stomach, intestine, peritoneum, kidney, and lung, was also decreased in Metavert and Saha treated mice, compared to Tideglusib or control treated mice, as shown in the representative images in **Fig. 1C**.

The results indicated that HDACs, but not GSK-3β, are responsible for PDAC metastasis in our model. Metavert and Saha inhibited both classes I and II HDACs. Therefore, we next determined which HDAC(s) are highly expressed in the tumors of patients with metastasis compared to those without metastasis. Analyzing the mRNA levels of HDACs 1 to 10 showed increased levels of all HDACs, except HDAC8, in the primary tumors of patients with metastasis compared to patients without metastasis (**Fig. 1D**). However, only HDACs 10 and 4 showed significance when comparing both the primary PDAC or the liver metastatic lesions of patients with metastasis with the primary PDAC of patients without metastasis (**Fig. 1D**). Next, we measured the protein levels of these HDACs in primary PDAC tissues and liver metastasis tissues from patients with and without metastasis. Immunohistochemical (IHC) staining of PDAC tissues from patients showed increased expression of HDACs 2, 3, 7 and 9 in tissues from primary PDAC and liver metastatic lesions of patients with metastasis compared to primary PDAC tissues of patients without metastasis (**Fig. 1E**). The other HDACs showed either similar level of expression or very weak to no expression in both groups of patients (**Fig. 1E**).

**Figure 2:**
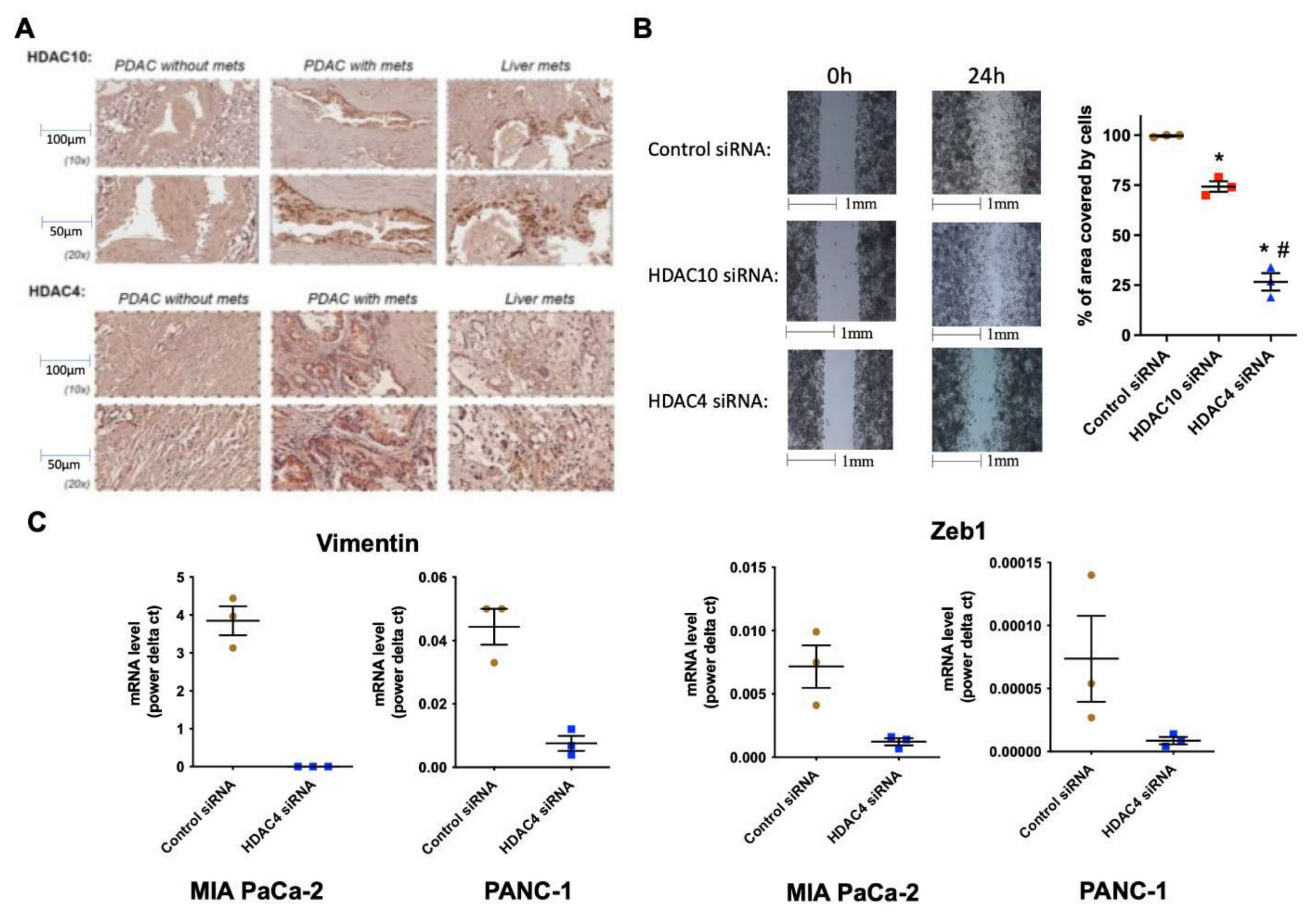
** HDAC4 and 10 are highly expressed in PDAC and liver metastases, and HDAC4 regulates PDAC cell migration and EMT.** IHC of HDAC10 & 4 in human PDAC and liver tissues (N=5) (A). Migration assay of MIA PaCa-2 cells transfected with HDAC10 & 4 siRNAs (B). mRNA levels of EMT markers in PDAC cells (C). *, *p* < 0.05 *versus* control siRNA. #, *p* < 0.05 *versus* HDAC10 siRNA.

Immunohistochemistry (IHC) of PDAC and liver tissues revealed robust expression of HDAC10 and HDAC4 in primary PDACs and metastatic liver lesions of patients with metastasis, compared to a weaker signal in tissues from patients without metastasis (**Fig. 2A**).

Metastatic cancer cells are characterized by an increased ability to migrate and undergo induced EMT. Molecular inhibition of both HDAC10 and HDAC4 in PDAC cells caused a decrease in migration (**Fig. 2B**). However, the effect with HDAC4 inhibition was statistically significant, suggesting that HDAC4 is likely a key regulator of migration. It is worth noting that HDAC10 and HDAC4 siRNAs did not alter the number of cells after 24 hours (not shown). In addition, silencing HDAC4 by siRNA significantly decreased the mRNA levels of EMT markers, such as vimentin and EMT transcription factor Zeb1, by over 80%, in MIA PaCa-2 and PANC-1 PDAC cells (**Fig. 2C**).

### HDAC4 and YAP overexpression are associated with worse outcomes in pancreatic cancer

**Figure 3:**
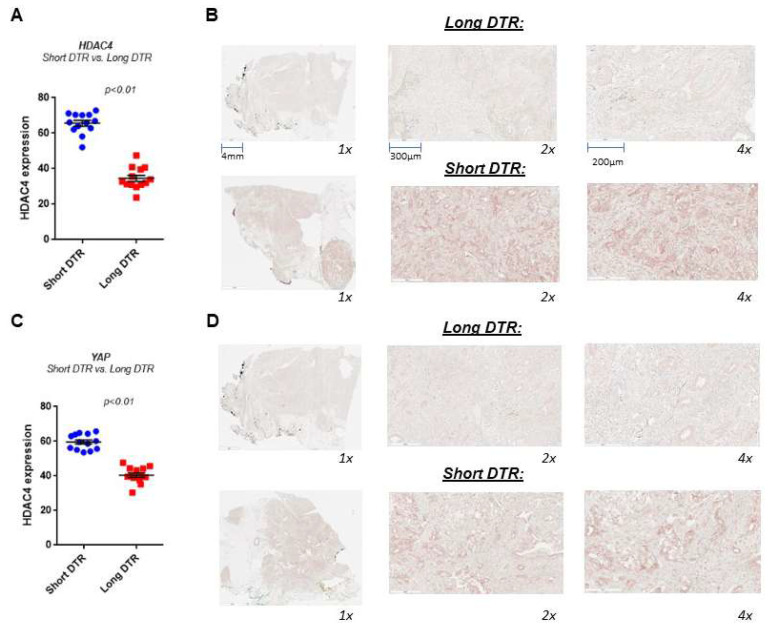
** HDAC4 and YAP are significantly highly expressed in PDAC tissues from patients with short days to recurrence (DTR) versus long DTR of metastasis.** IHC of HDAC4 and YAP in human PDAC tissues from patients with short and long DTR (N=13). Quantification of the staining *vs.* DTR (A, C). Representative photomicrographs of the stained tissues (B, D).

To examine whether HDAC4 and YAP levels correlate with clinical outcomes, we performed an analysis of the PDAC tissues collected from patients with various lengths of recurrence-free period, as measured by months from initial treatment to recurrence (DTRs) with the disease in metastasis sites. We define short DTR as recurrence within 6 months and long DTR as longer than 6 months. The tissues were collected from biopsies or at the time of surgery and before any adjuvant chemotherapy treatment. We found that patients with short DTR have 2-fold more of the HDAC4 protein expression in PDAC tissues compared to those with long DTR (**Fig. 3A, B**). Similarly, we found that YAP protein levels are also significantly higher in the tissues of patients with short DTRs compared to those with long DTRs (**Fig. 3C, D**). The association between the levels of HDAC4 and YAP and the rapid recurrence and metastasis of PDAC suggests that they may act on a common pathway to promote the disease.

### HDAC4/MybL1/YAP pathway regulates metastasis

**Figure 4:**
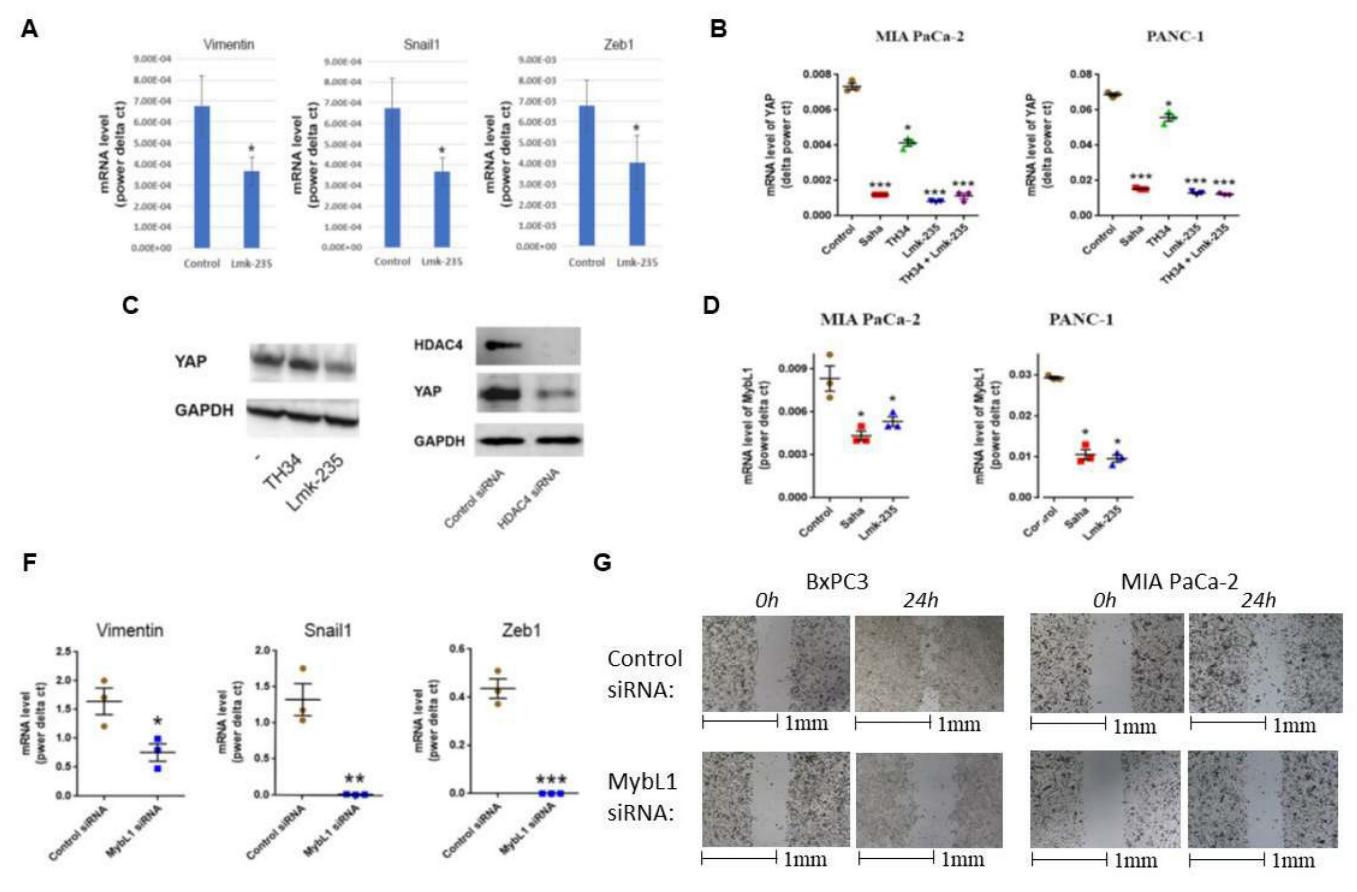
** HDAC4 regulates transcription factors MybL1 and YAP in PDAC cells.** mRNA level of Vimentin, Snail1, and Zeb1 in PDAC tumors from mice treated with Lmk-235 (3mg/kg) for 4 weeks (3 times/week) or vehicle (A). mRNA level of YAP (B) and MybL1 (E) in PDAC cells treated for 48h with Pan-HDAC inhibitor Saha (5µM), HDAC10 inhibitor TH34 (10µM), and HDAC4 inhibitor Lmk-235 (5µM) (B) or transfected with YAP or MybL1 plasmids. Protein level of YAP in PANC1 cancer cells treated with TH34 and Lmk235 (C) or transfected with HDAC4 or control siRNAs for 48h (D). mRNA levels of EMT markers in BxPC3 cancer cells 48h after transfection with MybL1 or control siRNAs (F). Migration assay of BxPC3 and PANC1 cancer cells transfected with MybL1 siRNAs for indicated times (G). *, *p* < 0.05 *versus* control. **, *p* < 0.01 *versus* control. ***, *p* < 0.005 *versus* control.

We examined the effect of HDAC4 inhibition on the levels of EMT markers in PDAC *in vivo*, using a syngeneic mouse model of PDAC, where UNKPC961-Luc PDAC cells were injected in the pancreas, followed by treated with HDAC4 inhibitor Lmk-235 or vehicle. We found that EMT markers vimentin, Snail1 and Zeb1 decreased significantly in mice treated with the HDAC4 inhibitor (**Fig. 4A**).

To determine the functional interaction between HDAC4 and YAP, we first inhibited HDAC4. We found that the HDAC4 inhibitor Lmk235 significantly decreased the level of YAP mRNA by 90% and 80% in MIA PaCa-2 and PANC-1 cells, respectively. In contrast, the HDAC10 inhibitor decreased the mRNA level of YAP by 40% and 25% in the same cell lines, respectively (**Fig. 4B**). YAP protein levels were significantly decreased by HDAC4 inhibition, but not by HDAC10 inhibition (**Fig. 4C**). Furthermore, HDAC4 siRNA reduced the protein level of YAP in PDAC cells (**Fig. 4D**). Importantly, we found that both the pan-HDAC inhibitor Saha and the HDAC4 specific inhibitor Lmk-235 significantly decreased the mRNA level of the transcription factor MybL1 in MIA PaCa-2 and PANC-1 cancer cells (**Fig. 4E**).

**Figure 5:**
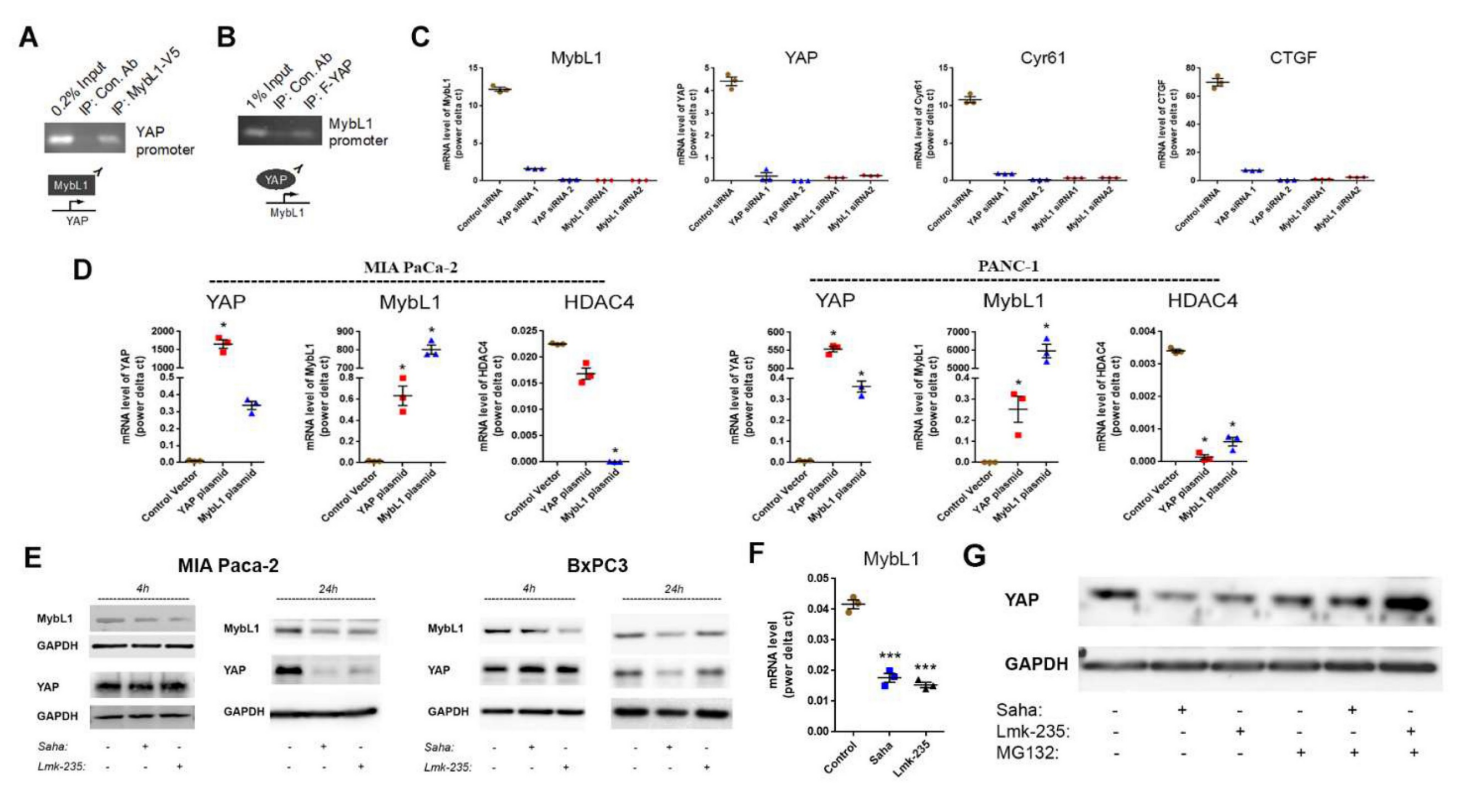
** MybL1 the mediates regulation of YAP expression by HDAC4.** Pull down assay using tagged MybL1 protein (A) and YAP tagged protein (B). mRNA levels of YAP, MybL1 and YAP targets Cyr61 and CTGF measured by RT-PCR 48h after transfecting cancer cells with YAP, MybL1 or control siRNAs (C). mRNA levels of YAP, MybL1 and HDAC4 measured by RT-PCR 48h after transfecting cancer cells with YAP or MybL1 overexpressing plasmids or control vector (D). Protein levels of YAP and MybL1 after 4h and 24h treatment of MIA and BxPC3 cells with HDAC4 or pan-HDACs inhibitors Lmk-235 and Saha (5µM) (E). Myb mRNA level 4h after treatment with Saha and Lmk-235 (5µM) (F). Protein levels of YAP after 24h treatment of MIA PaCa-2 cells with Lmk-235 and Saha (5µM) or MG132 (1µM) (G). *, *p* < 0.05 *versus* control.

We also identified MybL1 as a candidate for YAP-regulated genes. siRNA knockdown of YAP led to reduction of MybL1 mRNA levels (**Fig. 5C-D**). To determine the effect of MybL1 on EMT and migration, we transfected PDAC cells with MybL1 siRNA. We found a significant decrease in EMT markers vimentin, Snail1, and Zeb1 in PDAC cells (**Fig. 4F**). The decrease of these markers was 70%, 95%, and 100%, respectively (**Fig 4F**). Furthermore, MybL1 siRNA induced a significant decrease in PDAC cell migration (**Fig. 4G**).

There were no published findings linking HDAC4, MybL1 and YAP together; therefore, we tested the hypothesis that HDAC4 regulates YAP expression through the MybL1 transcription factor. Using the chromatin immunoprecipitation assay, we found that MybL1 binds to the YAP promoter (**Fig. 5A**), suggesting MybL1 may directly regulate transcription of YAP. Interestingly, we also found that YAP is present in the MybL1 promotor (**Fig. 5B**). When performing an immunoprecipitation of YAP in cells either with or without treatment with HDAC inhibitors, we did not observe any presence of HDAC4 or MybL1 (not shown), which suggests that these proteins may not interact with each other directly. The interdependent regulation between YAP and MybL1 was further confirmed using YAP and MybL1 siRNAs. YAP siRNA induced a decrease in the mRNA level of MybL1, whereas ablation of MyBL1 led downregulation of YAP (**Fig. 5C).** In addition, knockdown of either YAP or MybL1 decreased the expression of the YAP down-stream target genes Connective Tissue Growth Factor (CTGF) and Cysteine-Rich Angiogenic Inducer 61 (Cyr61) (**Fig. 5C).** Of note, both CTGF and Cyr61 are known to up-regulate cancer metastasis^26^, ^27^. Conversely, we found that MybL1 overexpression induced an increase in YAP mRNA level. Similarly, YAP overexpression induced an increase in MybL1 mRNA (**Fig. 5D**), indicating an inter-regulation of transcription between MybL1 and YAP. Furthermore, we found a decrease in the mRNA level of HDAC4 during over-expression of MybL1 or YAP, suggesting a negative feedback loop from YAP and MybL1 towards HDAC4 (**Fig. 5D**).

To determine which one of the two transcription factors, MybL1 and YAP, is regulated first by HDAC4, we measured the effect of pan-HDAC and HDAC4 inhibition on the protein levels of YAP and MybL1 over time. We found that a 4-hour treatment with the pan-HDACs or HDAC4 inhibitor in MIA PaCa-2 and BxPC3 cancer cells decreased the protein levels of MybL1. At the same time, those of YAP did not change (**Fig. 5E**). In contrast, after 24 hours of treatment with the HDAC inhibitors, both MybL1 and YAP protein levels were reduced (**Fig. 5E**). Treatment for 4 hours with HDAC inhibitors caused a decrease in MybL1 mRNA (**Fig. 5F**) confirming the transcriptional regulation of MybL1 by HDAC4. These results indicate that MybL1 is initially regulated by HDAC4, followed by regulation of YAP transcription. In addition to the transcriptional regulation of MybL1, we found that HDAC4 regulates YAP post-transcriptionally by inhibiting its degradation. Proteasome inhibitor MG132 induced an increase in the protein level of YAP in the presence of HDAC inhibitors for 24 hours (**Fig. 5G**). Furthermore, YAP and MybL1 staining of PDAC cells treated with HDAC4 and pan-HDAC inhibitors shows that there is a decrease in the expression of the two proteins, but without any significant changes in their localization between the cytoplasm and the nucleus (**Supplementary Fig. 1**).

To confirm that the regulation of YAP by HDAC/MybL1 is through chromatin remodeling, we performed ATAC-seq of MIA PaCa-2 cells treated with HDAC4 or pan-HDAC inhibitors Lmk-235 and Saha, respectively. Focusing on the YAP gene, strong ATAC-seq peaks were observed at the YAP promoter region in the control condition, indicating an open chromatin state and active transcription (**Supplementary Fig. 2A**). Lmk-235-treated cells displayed fewer and weaker peaks, suggesting partial chromatin compaction, while Saha-treated cells exhibited a near-complete loss of peaks, indicating chromatin closure and reduced accessibility (**Supplementary Fig. 2A**).

Within the YAP gene promoter region, we identified motifs for transcriptional factor binding sites under the control (**Supplementary Fig. 2B**), Lmk-235 treatment (**Supplementary Fig. 2C**), and Saha treatment (**Supplementary Fig. 2D**) conditions. To identify potential transcription factor binding, we selected the top-ranked motif from each condition. We performed a TOMTOM search against the HOCOMOCO transcription factor database, allowing us to establish a list of transcription factors associated with chromatin accessibility changes at the YAP promoter for Control (**Table 1**), Lmk-235 (**Table 2**), and Saha (**Table 3**). The top five transcription factors for the Control condition were SPIB, SPI1, FLI1, BCL11A, and IRF3, with their optimal alignments shown in **Supplementary Figure 3A**. SPIB has been seen to play a role in tumor suppression, metastasis, and chemosensitivity in colorectal cancer^28^ and lung cancer^29^. SPI1 is increased in pancreatic cancer and has been tied to the activation of the WNT signaling pathway, along with other cancer pathways^30^. FLI1 is upregulated in pancreatic cancer^31^, and is a player in mediating gemcitabine resistance in pancreatic cancer^32^, and has been linked to YAP signaling with regards to endothelial cell differentiation^33^. BCL11A has also been identified as a possible prognostic marker for pancreatic cancer, as it has been linked to poorer overall survival^34^. While IRF3 has been shown to indirectly enhance PDAC cell proliferation and invasion through a novel circular RNA axis^35^. For Lmk-235 treatment, the top five were SRY, ZNF143, GFI1B, GFI1, and ZNF85, with their optimal alignments shown in **Supplementary Figure 3B**, while in the Saha condition, the top five were FLI1, ETS2, VEZF1, ZNF418, and ZNF341, with their optimal alignments shown in **Supplementary Figure 3C**. The transcription factors in the Lmk-235 condition are mainly involved in transcriptional repression^36^, chromatin remodeling, and regulating cell differentiation and proliferation^37^. ZNF143, interestingly, has been seen to mediate the Hippo/YAP signaling pathway in glioma cells, causing cell growth and migration^38^. ZNF143 has also been seen to contribute to inflammation and proliferation of ovarian cancer^39^. On the other hand, the transcription factors in the Saha condition are mainly involved in angiogenesis, inflammatory signaling, vascular development, and immune regulation^40^. Among these transcription factors, ETS2 overexpression has been linked to the progression of pancreatic adenocarcinoma and associated with aggressive phenotypes, such as lymph node metastasis and vascular invasion^41^.

We assessed whether HDAC inhibition affects the accessibility to the potential MybL1 binding sites within the YAP promoter region. Using FIMO motif scanning, we identified multiple MybL1 binding sites. In the control condition, MybL1 motifs were highly enriched, with several significant motifs detected across accessible chromatin regions. The highest-scoring MybL1 motif (YAACKG) had a score of 5.22 and a p-value of 0.00182, with frequently matched sequences including CAACCG, AAACGG, and CGACGG (**Table 4**). In the Lmk-235 or Saha treatment conditions, the accessibility of these sites was significantly reduced. MybL1 motifs were still detected but with lower frequency and altered distribution. The top-ranked motif in Lmk-235-treated samples retained a high score of 5.22 (p-value = 0.00182), indicating that some MybL1 binding sites remain intact despite HDAC4 inhibition (**Table 5**). However, a notable reduction in the number of significant MybL1 motifs was observed. This confirms that HDAC4 inhibition reduces MybL1 accessibility at the YAP promoter region.

In contrast, the Saha-treated condition exhibited an intermediate effect, with MybL1 motifs still detectable but showing moderate reductions in accessibility compared to the control. The highest-scoring Saha-associated MybL1 motif (YAACKG) again yielded a score of 5.22 (p-value = 0.00182), and matched sequences included CAACCG, AAACGG, and CGACGG, similar to the control condition (**Table 6**). However, additional motif sequences such as TTACGG and CAAAGG were uniquely detected in the Saha-treated condition, suggesting a potential shift in MybL1 motif preferences or accessibility following treatment.

Similarly, at the MybL1 gene promoter region, chromatin accessibility was altered following HDAC4 or pan-HDAC inhibition (**Supplementary Fig. 4A**). In the control condition, robust ATAC-seq peaks were detected, indicating an open chromatin state and potential transcriptional activity. However, motif analysis revealed no significant transcription factor motifs within these peaks, suggesting that while the promoter remained accessible, TEAD (and by extension YAP-TEAD) transcription factor binding may not be strongly enriched at this site (**Supplementary Fig. 4B**). In contrast, Lmk- and Saha-treated cells exhibited a complete loss of promoter accessibility, with no detectable ATAC-seq peaks in the MybL1 promoter, making it impossible to assess YAP/TEAD binding within this region (**Supplementary Fig. 4A**). To further investigate potential regulators of MybL1, TEAD transcription factor motifs were analyzed within the accessible chromatin regions in the control condition. Several TEAD motifs (CATTCCW) were detected, with the highest-scoring TEAD motif had a score of 7 (p = 0.00127, q = 0.496), with most detected motifs showing moderate to low significance (q-values ranging from 0.496 to 0.575) (**Table 7**).

Together, these findings suggest that chromatin accessibility at the YAP promoter is reduced in cancer cells treated with HDAC4 or pan-HDAC inhibitors. Additionally, HDAC4 inhibition alters the binding potential of the MybL1 transcription factor within the YAP gene promoter regions. The loss of MybL1 binding motifs in treatment conditions aligns with the global changes in chromatin accessibility observed in ATAC-seq analysis, indicating that HDAC4 inhibition restricts MybL1 recruitment to the YAP promoter and regulatory regions, potentially contributing to YAP transcriptional suppression.

Notably, both YAP and MybL1 expression levels are linked to increased metastasis. Indeed, YAP and MybL1 levels were higher in PDAC tissue samples from patients with metastasis compared to patients without metastasis (**Supplementary Fig. 5**). Furthermore, HDAC4 is known to be a regulated transcription factor Sp1^3^. We found that the Sp1 protein level, as well as its localization in the nucleus, is increased in PDAC tissues from patients with metastasis compared to those without metastasis (**Supplementary Fig. 5**).

Importantly, we found that the HDAC4/MybL1/YAP pathway is also present in colon and prostate cancer cells, consistent with our observations in pancreatic cancer cells. We found that inhibition of HDAC4, but not HDAC10, down-regulates the mRNA level of YAP (**Supplementary Fig. 6A, D**) and MybL1 (**Supplementary Fig. 6C, F**) in prostate and colon cancer cells. We also found that pan-HDAC and HDAC4, but not HDAC10 inhibitors, decreased YAP protein levels in both cell types (**Supplementary Fig. 6B, E**).

### YAP regulates metastasis in PDAC metastatic models in mice

**Figure 6:**
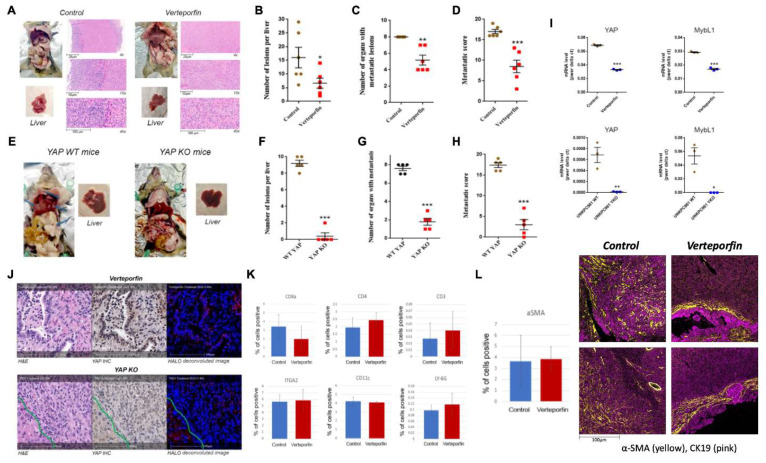
** YAP inhibition significantly reduces metastasis in mice with PDAC.** Metastatic mouse models of PDAC using syngeneic mice treated with Verteporfin or vehicle (A-D) or using UNKPC961-Luc- wild type and YAP KO cells (E-H). Quantification of the number of liver metastatic lesions (B, F), number of organs affected with lesions (C, G), and the metastatic score (D, H). mRNA level of YAP and MybL1 in UNKPC961 cells treated with or without 2µg/ml of Verteporfin and in UNKPC961 WT and YAP KO cells (I). H&E, IHC of YAP, and deconvoluted images of mouse liver tissues with PDAC metastatic lesions (J). In J, cells in the left corner of the YKO cells are hepatocytes with high YAP level. Percentage of positive cells for T cell markers, NK cells, dendritic cells and neutrophils in the liver were quantified (K). Percentage of positive SCs in the liver tissues was quantified and images of the distribution of SCs around the tumors in shown (L). *, *p* < 0.05, **, *p* < 0.01, and ***, *p* < 0.005 *versus* control.

Our data suggest that HDAC4 regulates PDAC metastasis by modulating MybL1, which in turn regulates YAP expression. We undertook two approaches to assess the effect of YAP inhibition on metastasis in mice. In the first approach, we used verteporfin, a photosensitizer for photodynamic therapy^42^ that previously was identified as an inhibitor of YAP^43^. Mice were injected with UNKPC961-Luc cells in the spleen to simulate liver metastasis, followed by treatment with Verteporfin (25mg/kg) for 6 weeks. We found that Verteporfin induced a significant decrease in the number of liver metastatic lesions by 60%, in the number of organs with metastatic lesions by 30%, and decreased the metastatic score by 50% (**Fig. 6A-D**). The metastatic score is based on the number and size of metastatic lesions observed in eight organs of the abdomen.

In the second approach, we developed UNKPC961-Luc cells with YAP knock out (YAP KO) and injected the cells in the spleen of mice. In this model, we found a significant decrease in the number of liver metastatic lesions by 90%, in the number of organs with metastatic lesions by 70%, and in the metastatic score by 80% in mice injected with YAP KO cells compared to mice injected with YAP wild type cells (**Fig. 6E-H**). Of note, both UNKPC961-Luc cells with WT and KO YAP grow at the same speed as shown by counting the number of cells for up to 15 days (**Supplementary Fig. 7A**).

Our IHC analysis of the liver tissues shows that Verteporfin treatment significantly decreased YAP protein levels in PDAC liver metastasis, and to a lesser extent, in the hepatocytes. However, not all PDAC cells had a significant decrease in YAP level as some of them maintained a level comparable to that in the hepatocytes. The liver tissues from mice injected with YAP KO UNKPC961 cells had very few metastatic lesions. The data clearly shows that YAP KO induced a much stronger effect on preventing metastasis compared to the pharmacological inhibition of YAP by Verteporfin. This is most likely due to the complete inhibition of YAP in KO cells, compared to a partial inhibition induced by Verteporfin. We found that Verteporfin induced a decrease in YAP target MybL1 as well as YAP mRNA levels by 40-50%. In contrast, YAP KO cells showed a reduction in YAP and MybL1 mRNA levels by over 99% (**Fig. 6I**). Importantly, IHC staining showed that YAP is undetectable in the tumors inoculated with UNKPC961 YAP KO cells (**Fig. 6J**). In contrast, in Verteporfin-treated mice, tumors exhibit a limited presence of YAP in the nuclei of cancer cells (**Fig. 6J**), indicating that YAP activation is not completely blocked.

Notably, the UNKPC961-Luc YAP KO cells exhibit a significant decrease of over 85% in EMT markers, including Vimentin, Snail1, and Zeb1 (**Supplementary Fig. 7B**), as well as a substantial decrease in migration (**Supplementary Fig. 7C**) compared to UNKPC961-Luc cells with wild-type YAP. IMC data showed no significant changes in the levels of multiple immune cells, such as T cells (CD8a, CD4, and CD3 positive cells), Natural Killer (NK) cells (ITGA2 positive), dendritic cells (CD11c positive cells), and neutrophils (LY-6G positive cells) between control and the Verteporfin treatment condition in the liver of mice (**Fig. 6K**). Importantly, we did not observe any change in the total number of tumor-associated hepatic stellate cells (HSCs) as indicated by the alpha-SMA staining quantification (**Fig. 6L**). However, analysis of the liver tissues from mice treated with Verteporfin showed a change in the distribution of HSCs in the tumor microenvironment. The HSCs were scattered throughout the tumor in control mice, but in the Verteporfin-treated mice, HSCs were primarily detected in regions that surround tumor cells (**Fig. 6L**). This is a fascinating observation, as previously published data showed the anti-cancer role of SCs in PDAC progression^44^.

**Figure 7:**
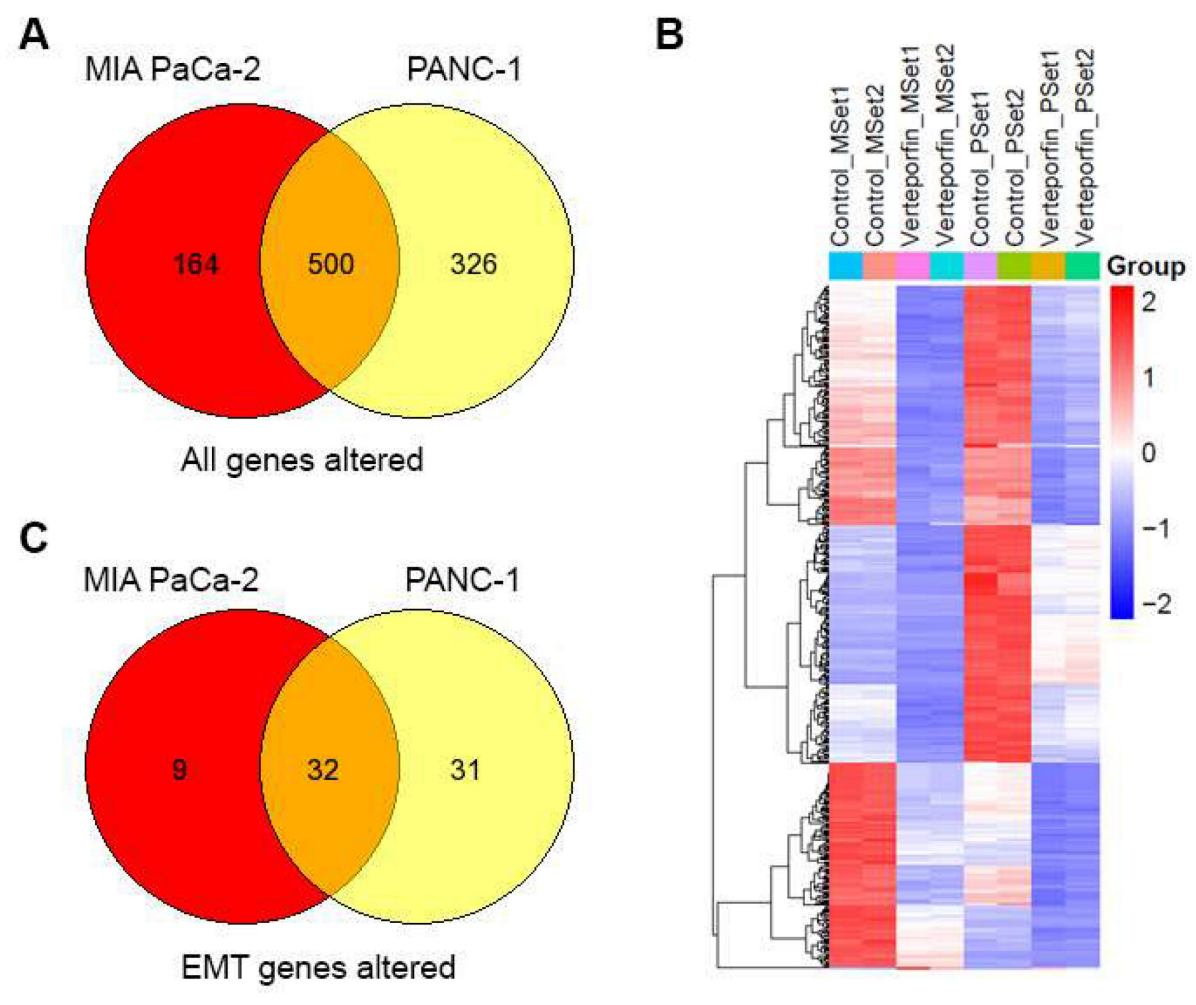
** YAP inhibition downregulates the EMT pathway in PDAC cells.** Overlap of genes with differential expression in MIA PaCa-2 and PANC-1 cells (A). Heatmap of n=2 replicates for differential expression (B). EMT pathway genes that overlap in differential expression between Mia PACA and PANC-1 cells (C).

To understand the molecular changes occurring in PDAC cells when they undergo treatment with Verteporfin, RNA-seq analysis was conducted on MIA PaCa-2 and PANC-1 cells after 18-24 hours of treatment. We observed that the expression of a sizable number of genes was downregulated in response to this treatment, specifically PANC-1 cells had 818 genes downregulated and 8 upregulated, while MIA PaCa-2 cells had 658 downregulated and 6 upregulated. Regardless of the cell line considered, 499 genes were downregulated, and HSPA6 was the only gene upregulated in both cell lines, as shown in the Venn Diagram (**Fig. 7A**) and heatmap of in RPKM (Reads Per Kilobase per Million) values (**Fig. 7B**).

Using pathway mapping to the Kyoto Encyclopedia of Genes and Genomes (KEGG) database^45^ combined with functional network reconstruction using semantic-based algorithms, we found that Epithelial to Mesenchymal Transition (EMT, MSigDB_Hallmarks) is enriched in each of the cell lines. For MIA PaCa-2 cells, the EMT pathway exhibits a q2 enrichment at 1.23E-20, with 41 of the 197 genes are present. For PANC-1 cells, the EMT pathway exhibits a q2 enrichment at 2.80E-38, with 63 of the 197 genes are present. While each cell line exhibits unique features for these pathways, 32 common genes are identified (**Fig. 7C**), and those specific to EMT pathways are listed in **Table 8**. Subsequently, we performed an upstream regulatory analysis to identify the type of transcriptional factors that regulate these genes. For this purpose, we mapped the most significant cis-regulatory domains for all known transcription factors onto the promoter of the EMT genes and found that they are directly connected to the function of AP-2 transcription factors, particularly TFAP2C. TFAP2A is a component of the ZEB1/2 network^46^, which is consistent with data shown in (**Fig. 2C, Fig 7F and Supplementary Fig 7B**). The AP-2 family of transcription factors is composed of five members, TFAP2A, TFAP2B, TFAP2C, TFAP2D, and TFAP2E, which are known to be involved in the regulation of EMT^47^. Thus, both the transcriptional network analyses along with upstream regulator mapping are congruent with the notion that EMT in our system is down regulated at the transcriptional level, making it a long-term response to Verteporfin. The UPR diagram in **Supplementary Fig. 8** shows the interaction between the pathways affected by Verteporfin.

## Discussion

In this study, we show that HDAC4 plays a critical role in metastasis and determine the mechanism of this role in PDAC metastasis.

We previously demonstrated the anti-metastasis effect of Metavert, a novel dual inhibitor of GSK-3β and HDACs^2^, ^48^ that targets all members of the HDAC families I and II including HDACs 1 to 10. Here, we found that treatment with an HDAC inhibitor, but not a GSK-3β inhibitor, is sufficient to inhibit PDAC metastasis. Analysis of tissues from PDAC patients revealed that those with metastasis exhibit a higher expression of most histone deacetylases compared to patients without metastasis. Importantly, HDAC4 and HDAC10 mRNA levels were significantly upregulated in both the primary PDAC tumors and the liver metastatic lesions of patients with metastasis compared to the primary tumors of patients without metastasis.

The protein levels of HDACs, particularly those of HDAC4, were also increased, as determined by IHC. Importantly, our analysis of the PDAC tissues from patients with short or long DTRs after chemotherapy showed that high levels of HDAC4 expression correlate with earlier recurrence. We noted that HDAC4 has been reported to be transcriptionally regulated by Sp1^3^. Interestingly, Sp1 was shown to be associated with increased metastasis in PDAC patients, while patients with no metastasis lacked the expression of sp1^4^.

Our *in vitro* study showed that inhibition of HDAC4 significantly reduced EMT and migration of PDAC cells, whereas inhibition of HDAC10 had little effect. Indeed, we have previously shown involvement of HDAC4 in regulating metastasis in the context of smoking-induced metastasis in a mouse model of PDAC with no insight on the mechanism^49^. Together, these findings suggest that HDAC4 may play a crucial role in mediating PDAC metastasis to the liver.

Our data indicate that HDAC4 can regulate YAP at both mRNA and protein levels. To our knowledge, this is the first study to demonstrate the transcriptional regulation of YAP by HDAC4. This event is mediated through a mechanism involving a positive feedback regulation loop between YAP and MybL1, where the two proteins interdependently regulate each other at the transcriptional level. Our data support the notion that molecular inhibition of HDAC4 first leads to downregulation of MybL1 through a yet-to-be-identified mechanism, which in turn results in the downregulation of both YAP and MybL1 mRNA levels. Surprisingly, the overexpression of YAP or MybL1 induced a decrease in HDAC4 level, indicating a possible negative feedback mechanism. Our data indicates that HDAC4 regulates MybL1 expression first, which is followed by a regulation of YAP transcription by MybL1. This represents a novel mechanism by which HDAC4 regulates YAP expression. Our ATAC-seq analysis supports the notion that HDAC4 inhibition, or HDAC inhibition in general, decreases the accessibility of MybL1 to its binding sites in the YAP promoter region.

We found that the HDAC4-MybL1-YAP signaling axis is involved in the expression of several EMT genes. YAP is a known regulator of EMT in many cancers^50^, ^51^. Our RNAseq data showed that the YAP inhibitor verteporfin significantly reduced the expression levels of a variety of EMT genes in PDAC cell lines.

A recent study showed that HDAC4 can modulate chemoresistance through a mechanism that involves activating YAP-mediated gene transcription and decreasing YAP phosphorylation at S127^52^, a site implicated in down regulation of YAP via proteasomal degradation or cytoplasmic retention. Consistent with this report, we found that YAP can be regulated post-transcriptionally by HDAC4, as inhibition of proteasomes prevented the decrease in YAP protein level induced by HDAC4 inhibition. Although the previous study showed that, when overexpressed, HDAC4 can physically bind to YAP^52^, we have not been able to detect such interactions at the endogenous level. In addition, our attempts on determining the effects of HDAC inhibition on the nuclear localization and the acetylation levels of either YAP or mybL1 did not provide any conclusive results.

This is the first study showing that YAP pharmacological and molecular inhibition can reduce liver metastasis using specific PDAC metastatic mouse models. The animal studies performed here demonstrate a direct effect of YAP on metastasis in pancreatic cancer using a splenic metastatic model of PDAC. Notably, YAP KO in PDAC cells decreased metastasis in mice. Consistent with this finding, we found that verteporfin, which was identified as an inhibitor of YAP^43^, significantly decreased PDAC metastasis to the liver and to other organs. The genetic inhibition of YAP induced a stronger effect to reduce metastasis compared to the pharmacological drug verteporfin. This is possibly due to effective ablation of YAP in YAP KO cells, compared to a partial inhibition of YAP by verteporfin. This notion is supported by our RNAseq data showing limited impact of verteporfin on the downstream targets of YAP. Moreover, verteporfin exhibits YAP-independent cytotoxic effects^53^. It is conceivable that the advent of YAP inhibitors with higher specificity and potency may lead to better therapeutic effects.

Our data indicates that verteporfin treatment did not change the level of the immune cells detected in the tumor microenvironment, including T cells, NK cells, dendritic cells, and neutrophils. However, although the overall level of SCs did not change significantly by verteporfin, the treatment altered the distribution of SCs in the liver metastatic lesions to the tumor boundary. This is important knowing that SCs were previously shown to play both pro and anti- cancer roles^44^, ^54^. Our results suggest that YAP inhibition may help SCs defend the organ against invasion by surrounding the tumors. Thus, YAP inhibition may promote the anti-cancer role of SCs.

In summary, our work demonstrated a novel interaction between HDAC4 and the transcription factors MybL1 and YAP. Clearly, a more detailed analysis of this functional interaction is needed. Moreover, our data showed that the key mediators of the metastasis pathway identified in pancreatic cancer are also present in colon and prostate cancers, but future investigations in other cancers will improve our understating of how important this pathway is in regulating metastasis in cancer in general. Nevertheless, the results of the current study provide insight into novel cellular and molecular mechanisms involving HDAC4, MybL1, and YAP leading to the regulation of metastasis. This is a novel cell signaling pathway identified with significant biomedical relevance.

## Supplementary Material

Supplementary figures and tables.

## Figures and Tables

**Table 1:**
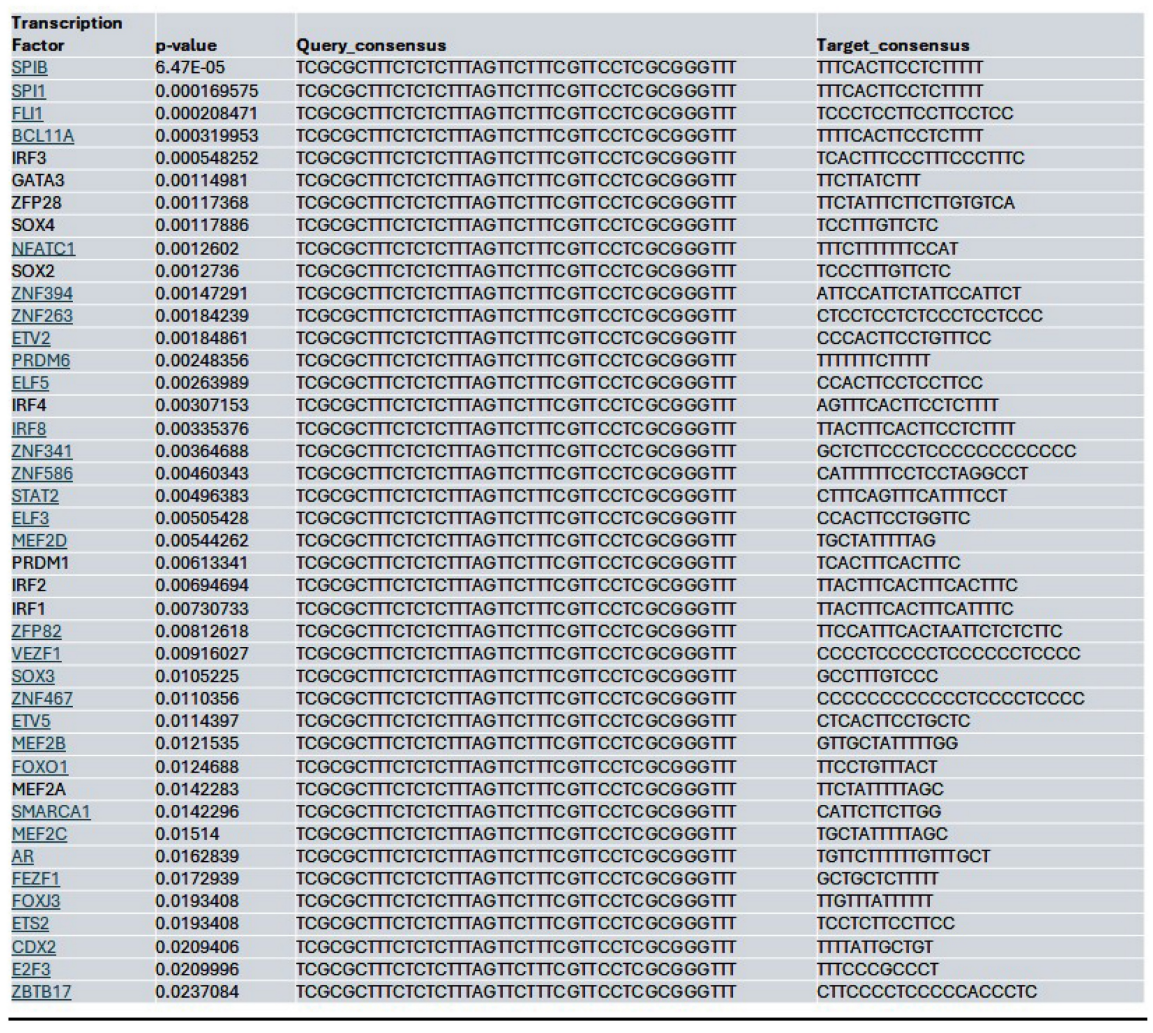
List of the top significant transcription factors associated with chromatin accessibility changes at the YAP promoter region for the control condition. Columns indicate the transcription factor, p-value, and the aligned query and target consensus sequences.

**Table 2:**
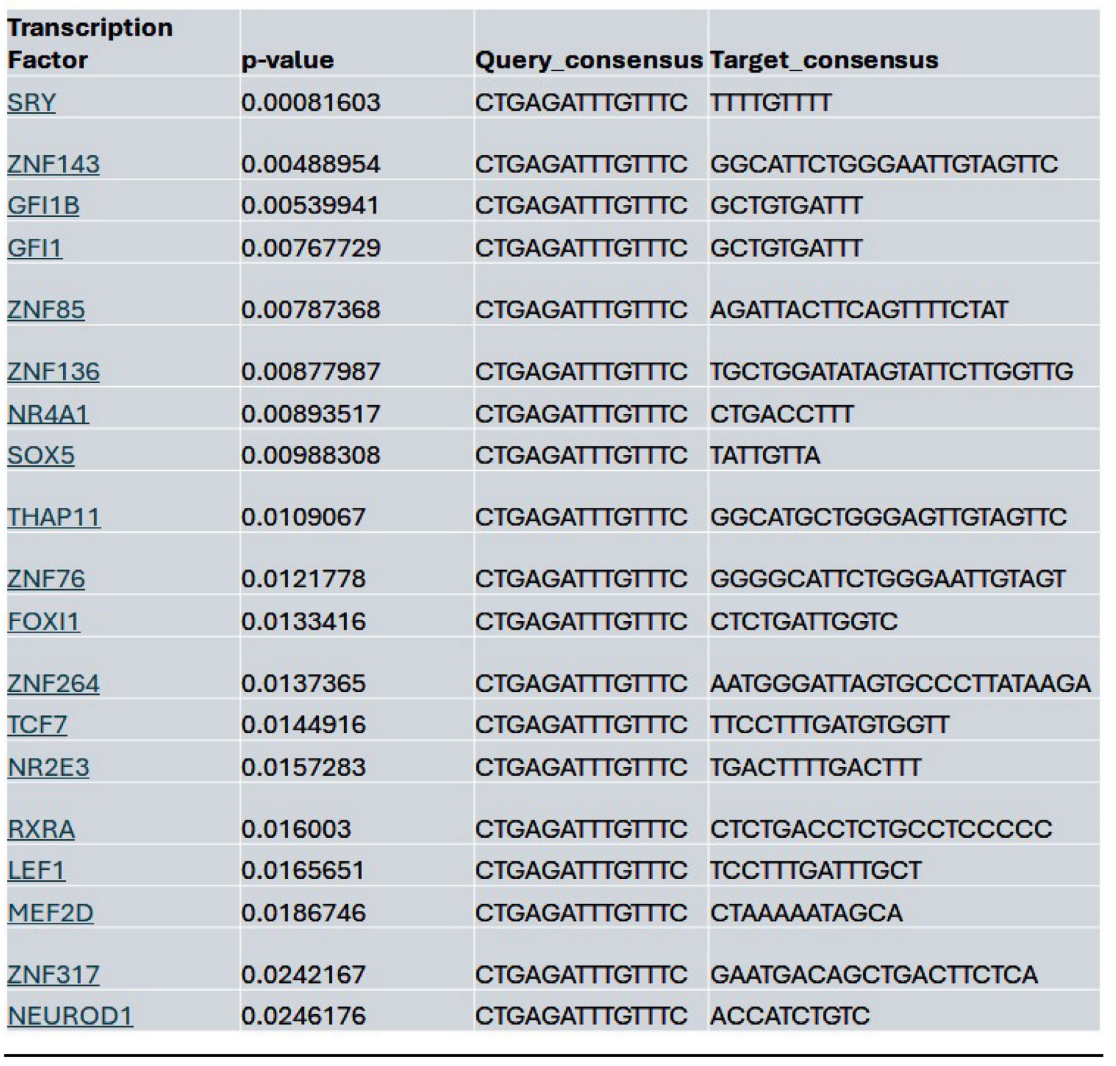
List of the top significant transcription factors associated with chromatin accessibility changes at the YAP promoter region for the Lmk-235 treated condition. Columns indicate the transcription factor, p-value, and the aligned query and target consensus sequences.

**Table 3:**
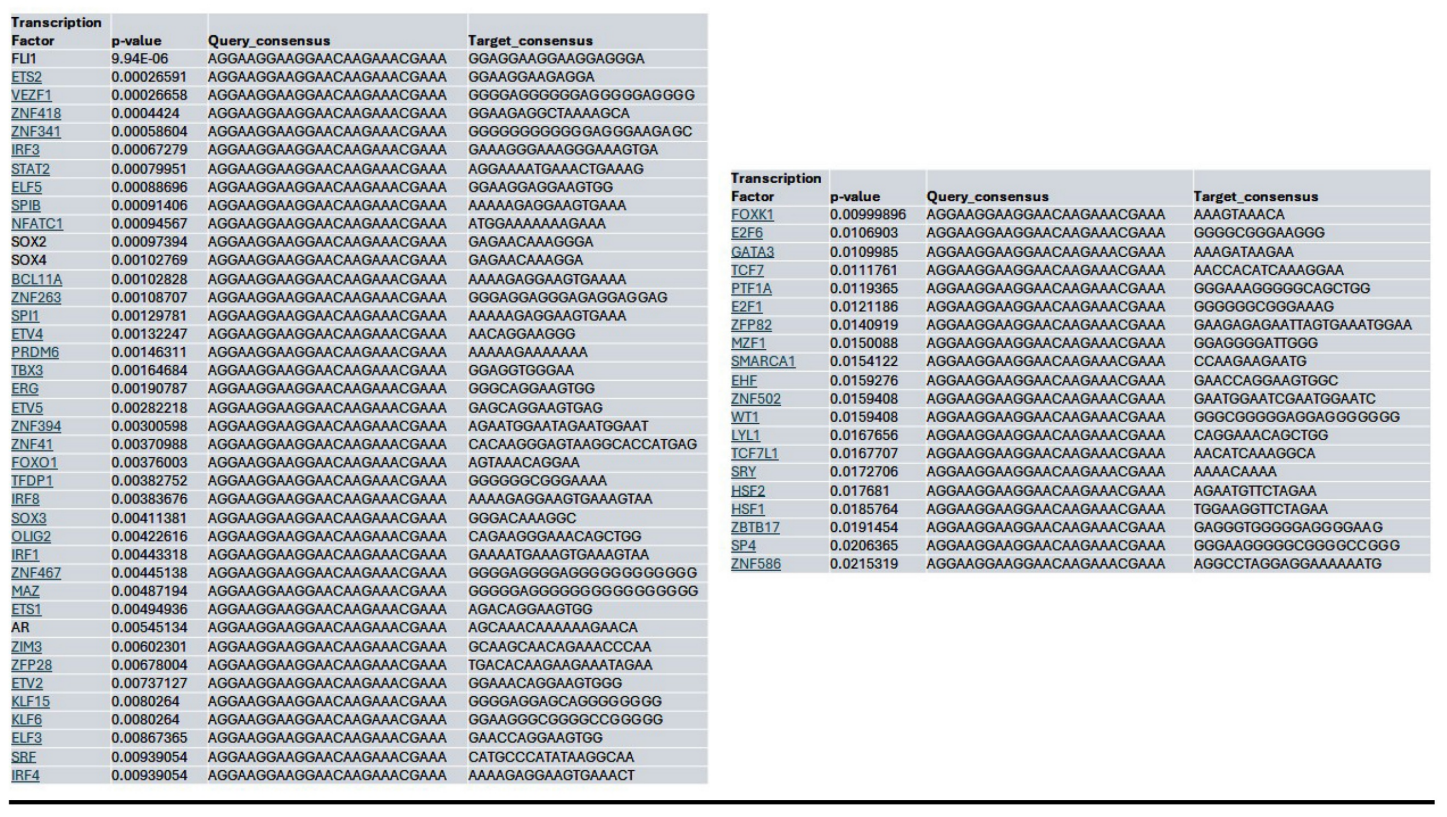
List of the top significant transcription factors associated with chromatin accessibility changes at the YAP promoter region for the Saha treated condition. Columns indicate the transcription factor, p-value, and the aligned query and target consensus sequences.

**Table 4:**
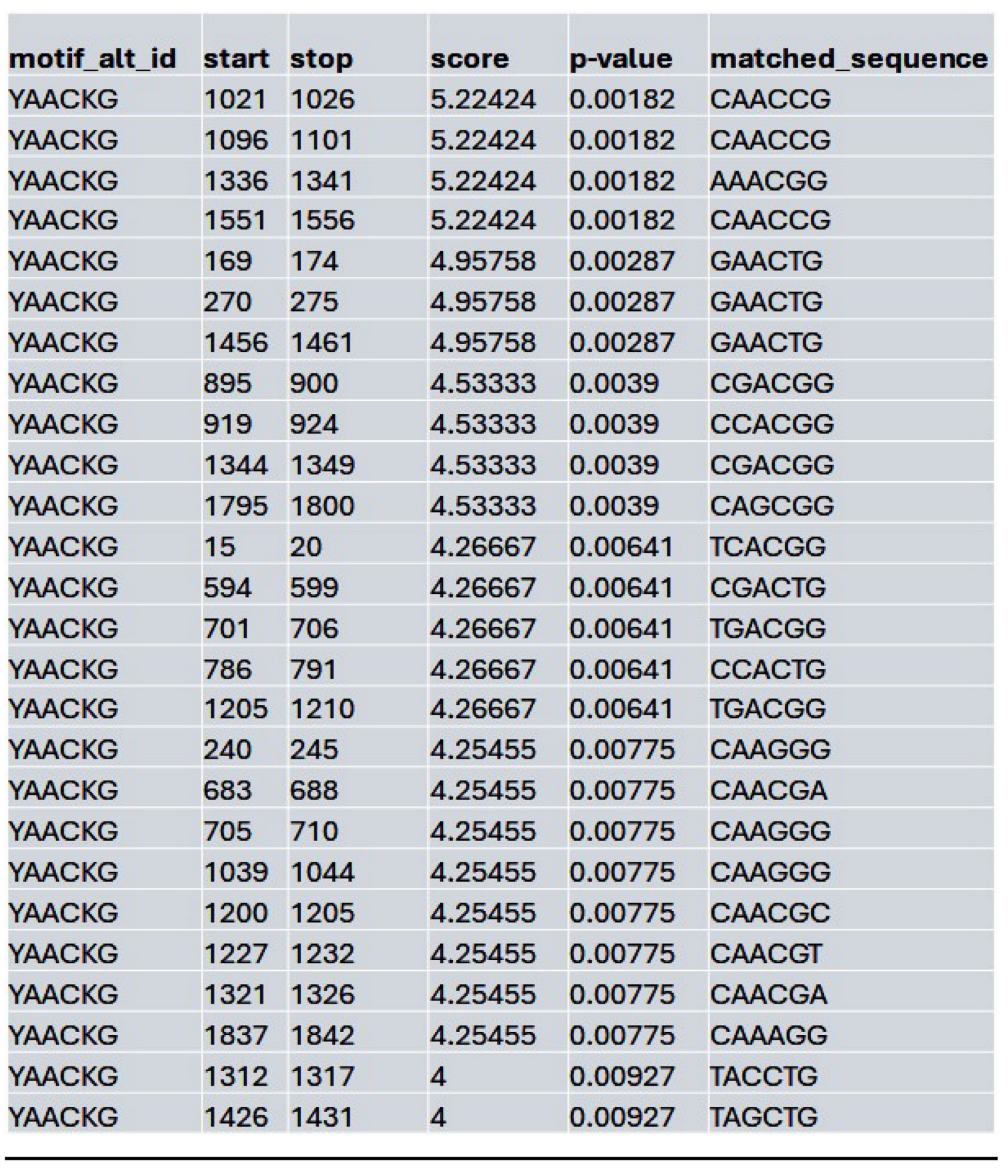
List of the top matched sequences to the MybL1 motif (YAACKG) in the control condition. Columns indicate the motif ID, start and stop positions of the matched sequence within the region, the motif match score, p-value, and the exact matched sequence.

**Table 5:**
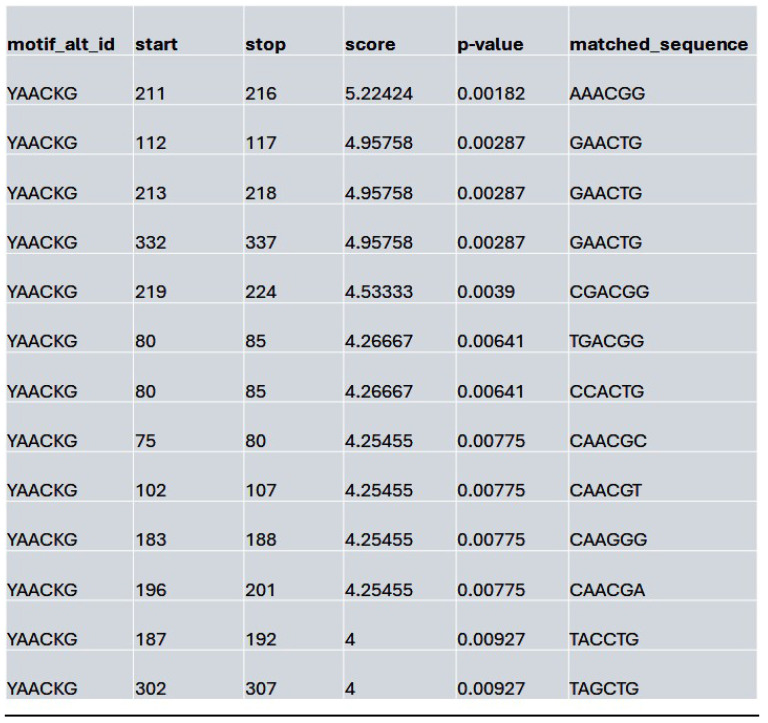
List of the top matched sequences to the MybL1 motif (YAACKG) in the Lmk-235 treated condition. Columns indicate the motif ID, start and stop positions of the matched sequence within the region, the motif match score, p-value, and the exact matched sequence.

**Table 6:**
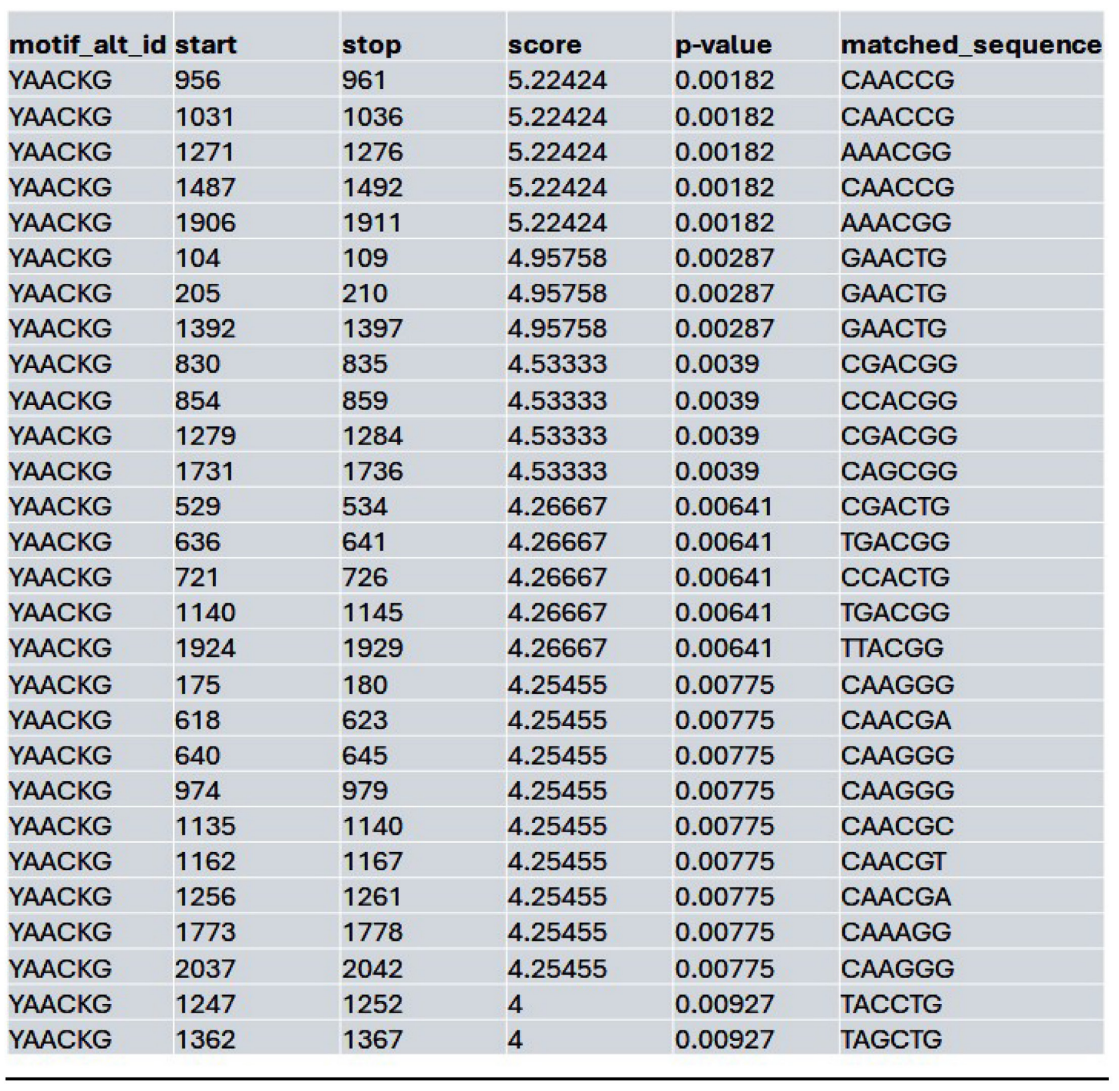
List of the top matched sequences to the MybL1 motif (YAACKG) in the Saha treated condition. Columns indicate the motif ID, start and stop positions of the matched sequence within the region, the motif match score, p-value, and the exact matched sequence.

**Table 7:**
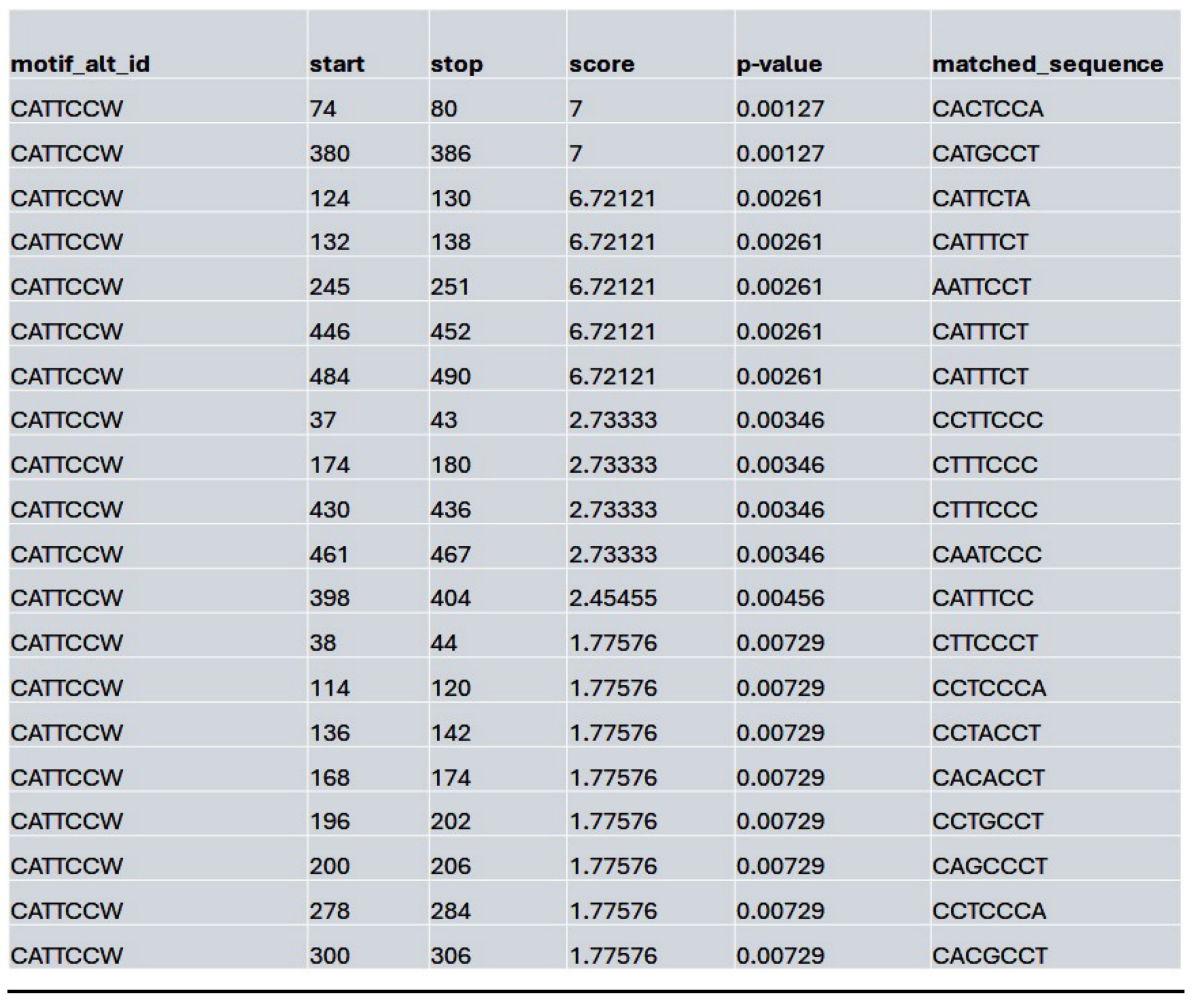
List of the top matched sequences to the TEAD-associated motif (CATTCCW) in the control condition. Columns indicate the motif ID, start and stop positions of the matched sequence within the region, the motif match score, p-value, and the exact matched sequence.

**Table 8:**
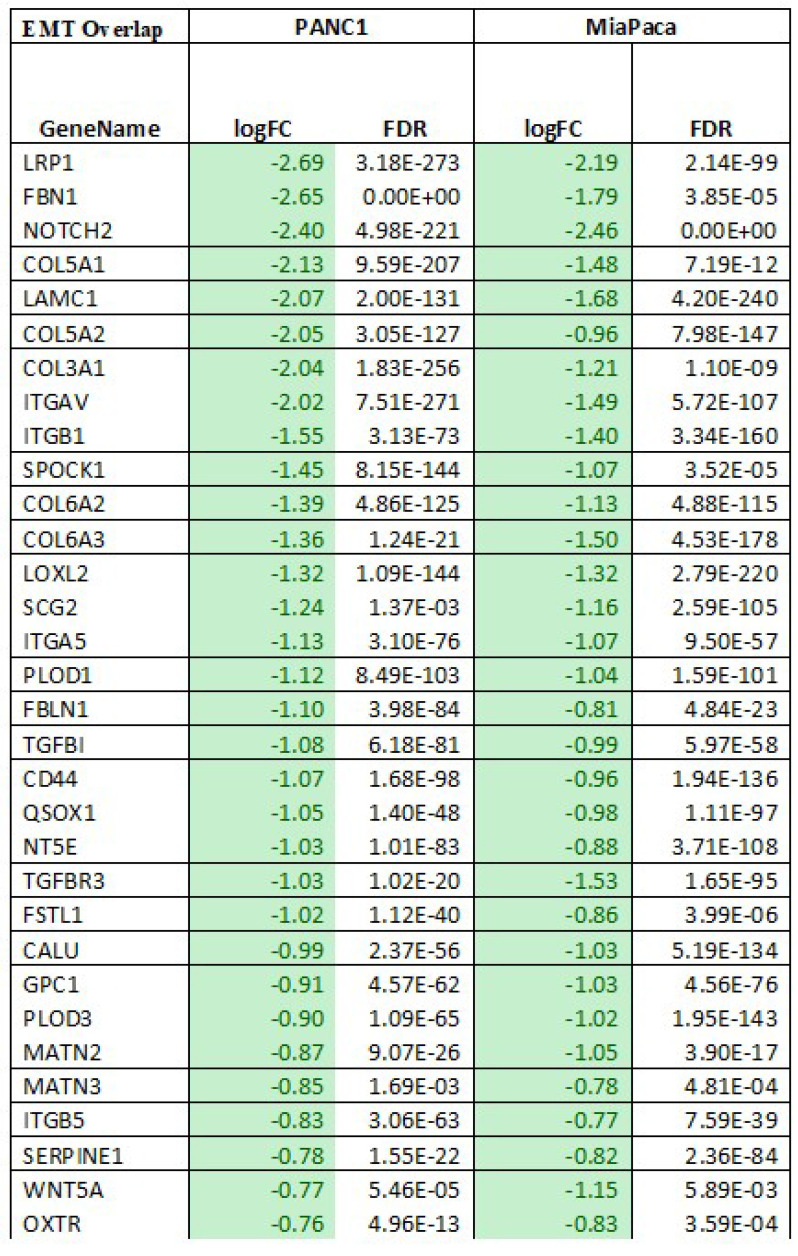
List of commonly differentiated genes associated with the EMT pathway in PANC-1 and MIA PaCa-2 cell lines. Columns show the gene name, log fold change (logFC), and false discovery rate (FDR).
