# Supplementary material for: HDAC4/MybL1/YAP novel signaling axis is required for pancreatic cancer metastasis to the liver
Source: Int J Biol Sci. 2025 Oct 24;21(15):6907–25. doi: 10.7150/ijbs.102132 (PMC12631223; doi:10.7150/ijbs.102132)
Supplement: Supplementary file 1 — Supplementary figures and tables. [file ijbsv21p6907s1.pdf]

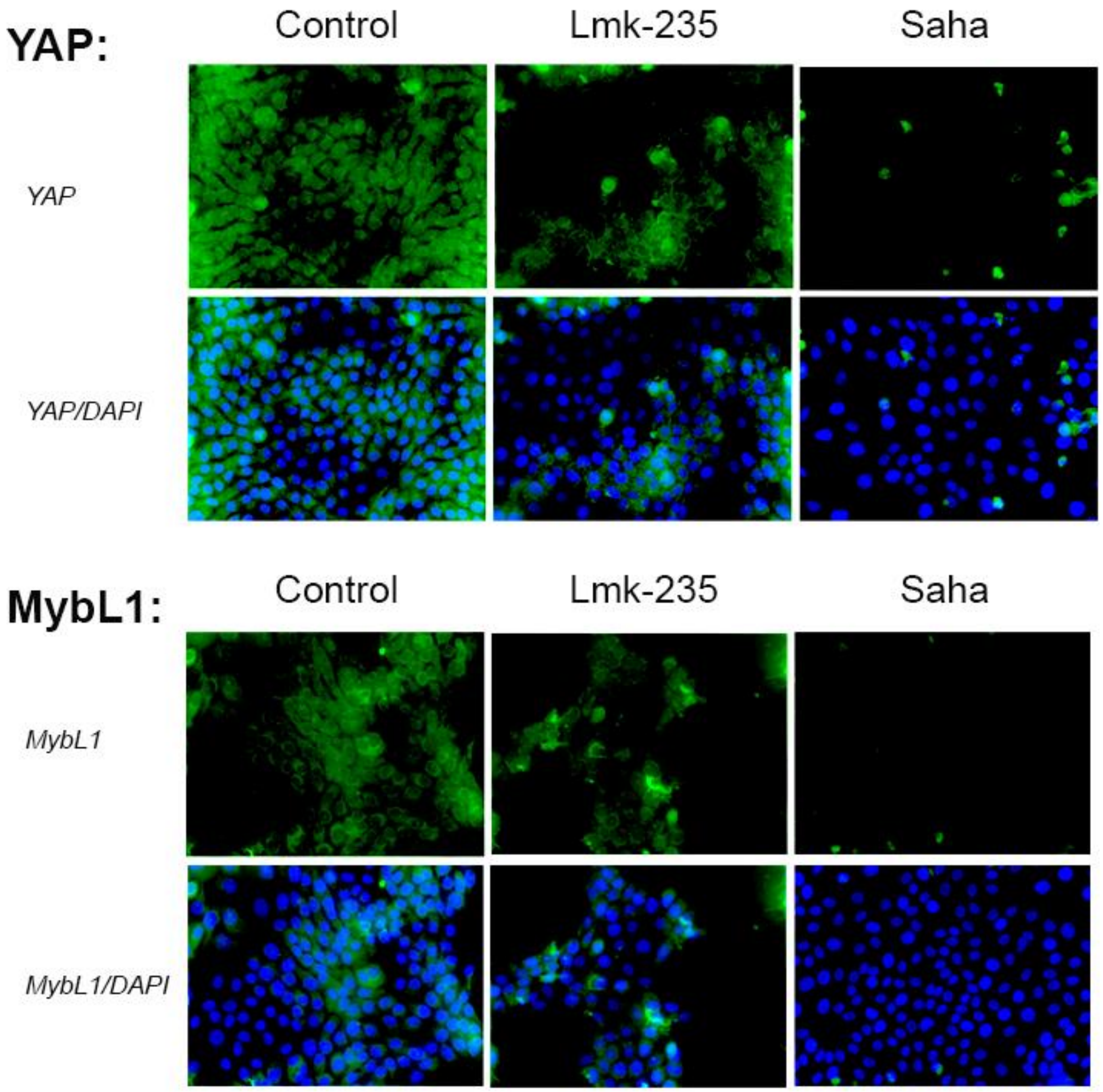

<u>Suppl Fig 1.</u> Total levels of YAP and MybL1 are decreased, but their localization is not affected by HDAC inhibition.

MIA PaCa-2 cells were treated for 24h with Pan-HDAC inhibitor Saha (5µM) or HDAC4 inhibitor Lmk-235 (5µM). Immunofluorescence staining of YAP and MybL1 with DAPI staining is shown.

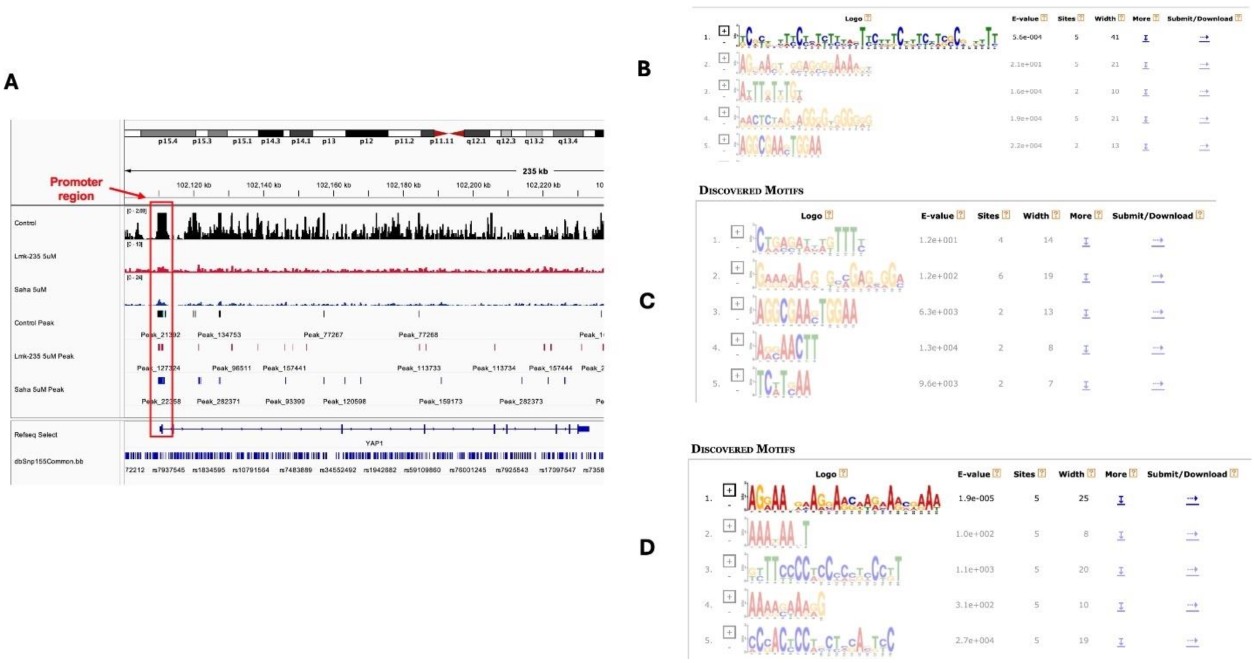

<u>Suppl Fig 2.</u> MIA PaCa-2 cells were treated with HDAC4 inhibitor Lmk-235 (5µM) or pan-HDAC inhibitor saha (5µM) for 48 hours and were used to perform ATAC-seq analysis. ATAC-seq peaks at the YAP promoter region in the control, Lmk-235 and Saha conditions (**A**). Identified motifs for the control (**B**), Lmk-235 treatment (**C**), and Saha treatment (**D**) conditions.

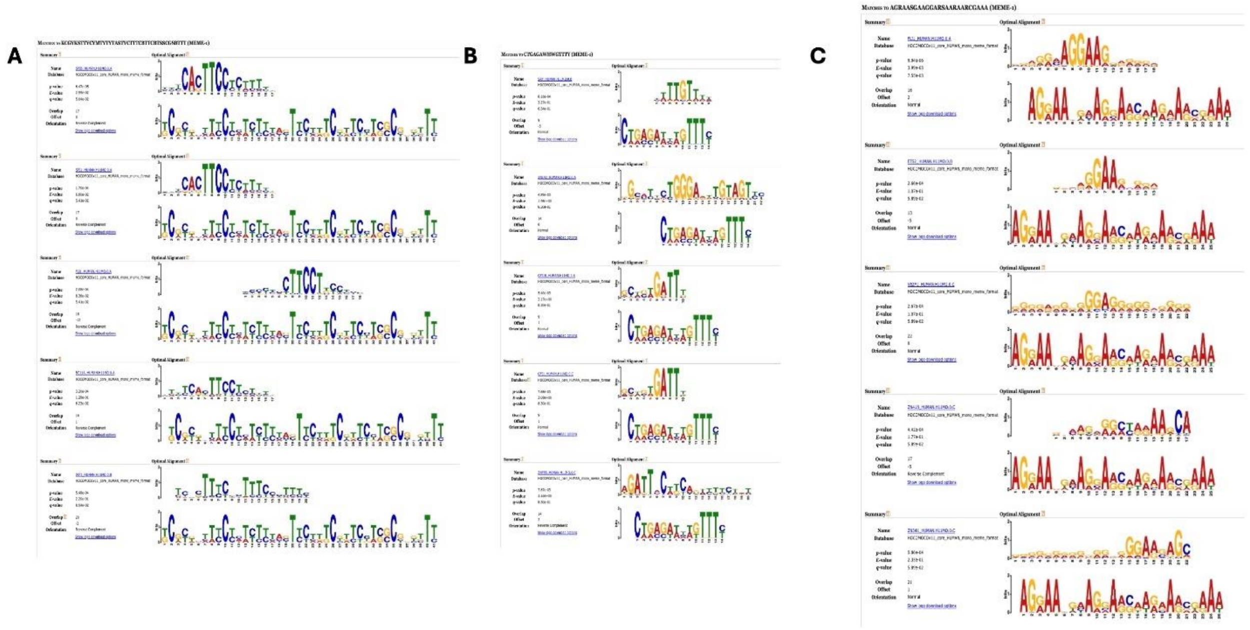

<u>Suppl Fig 3.</u> MIA PaCa-2 cells were treated with HDAC4 inhibitor Lmk-235 (5µM) or pan-HDAC inhibitor Saha (5µM) for 48 hours and and were used to perform ATAC-seq analysis. The top five transcription factors for the Control (**A**), Lmk-235 (**B**), and Saha condition (**C**) with their optimal alignments are shown.

A

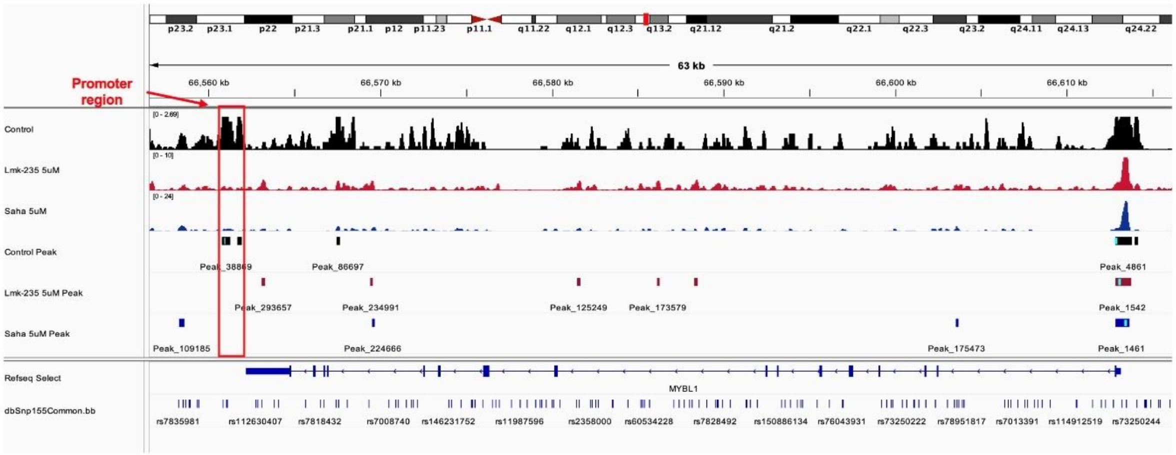

B

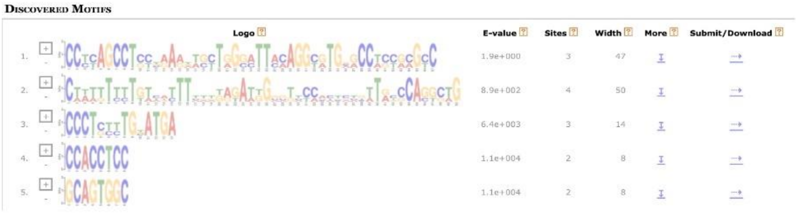

Suppl Fig 4. MIA PaCa-2 cells were treated with HDAC4 inhibitor Lmk-235 (5µM) or pan-HDAC inhibitor Saha (5µM) for 48 hours and and were used to perform ATAC-seq analysis. ATAC-seq peaks at the MybL1 promoter region in the control, Lmk-235 and Saha conditions (**A**). Identified motifs for the control condition (**B**).

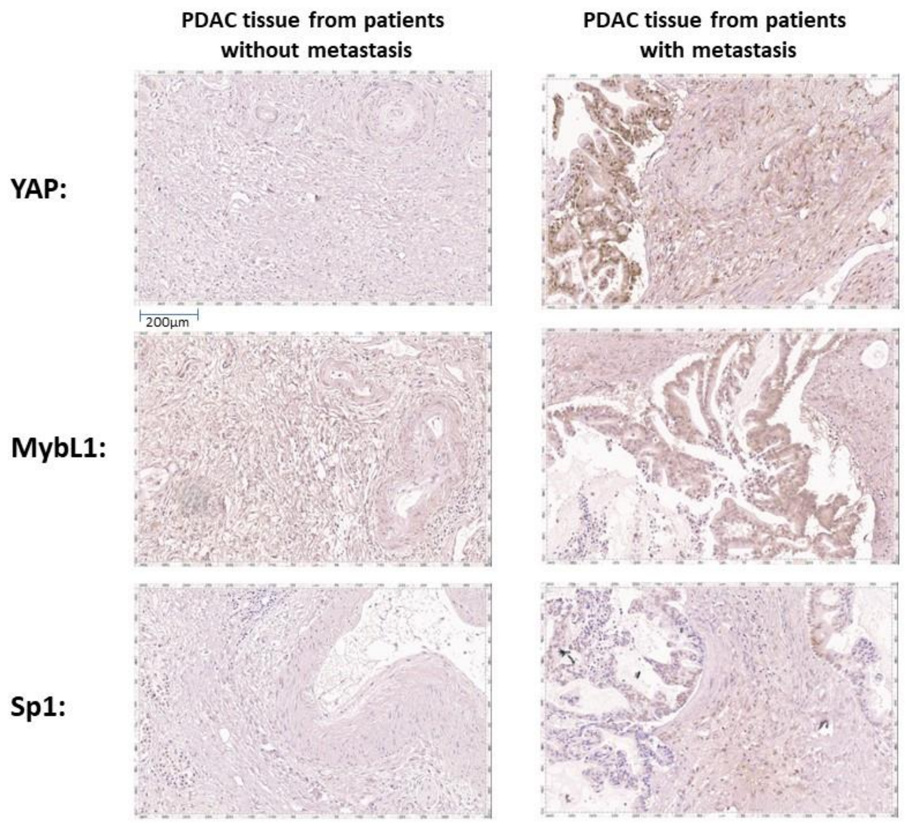

<u>Suppl Fig 5.</u> YAP, MybL1, and Sp1 are highly expressed in PDAC tissues from patient with liver metastasis compared to patients without metastasis.

IHC of YAP, MybL1, and Sp1 in human PDAC tissues (N=5).

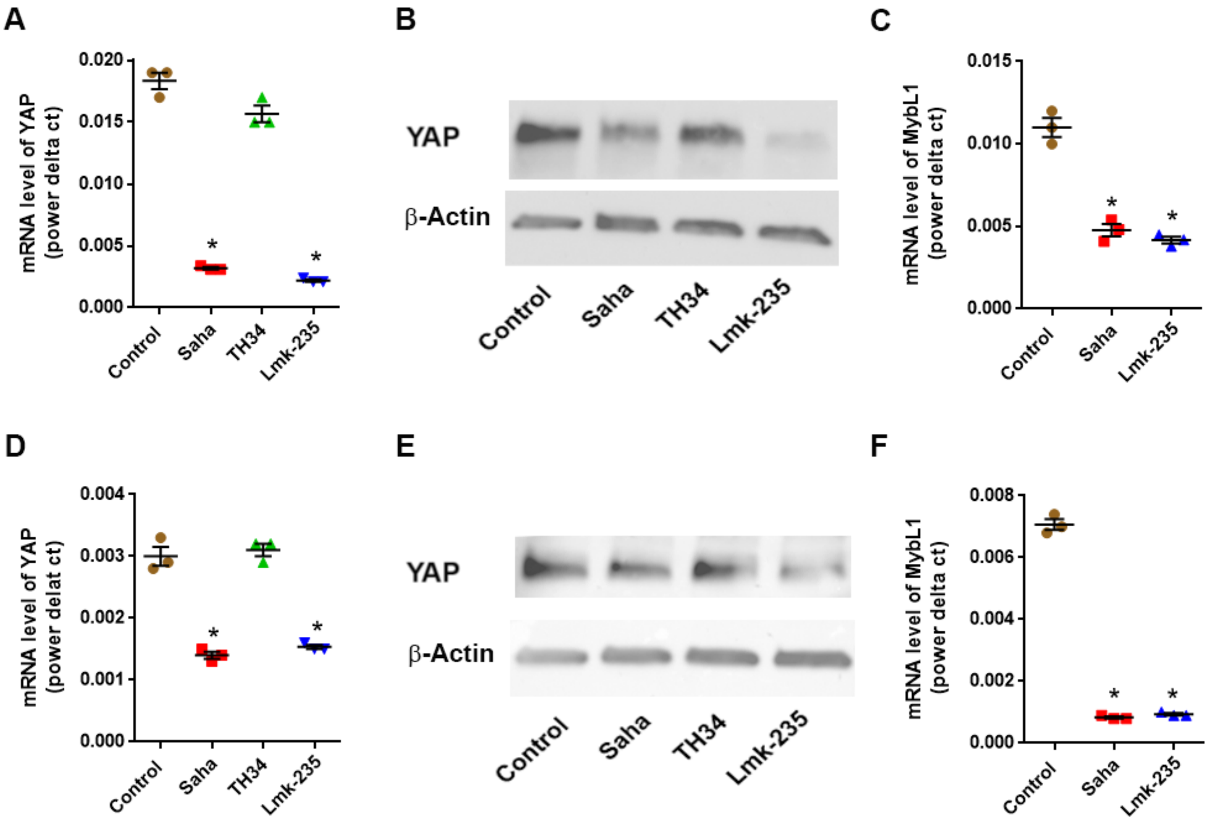

<u>Suppl Fig 6.</u> HDAC4 regulates MybL1 and YAP expression in colon and prostate cancer cells.

mRNA level of YAP (A, D) and MybL1 (C, F) in colon cancer cells RKO (A-C) and prostate cancer cells 22rv1 (D-F) treated for 48h with Pan-HDAC inhibitor Saha (5µM), HDAC10 inhibitor TH34 (10µM), and HDAC4 inhibitor Lmk-235 (5µM). Protein level of YAP in RKO cancer cells (B) and in 22rv1 cancer cells (E). *, $p < 0.05$ *versus* control.

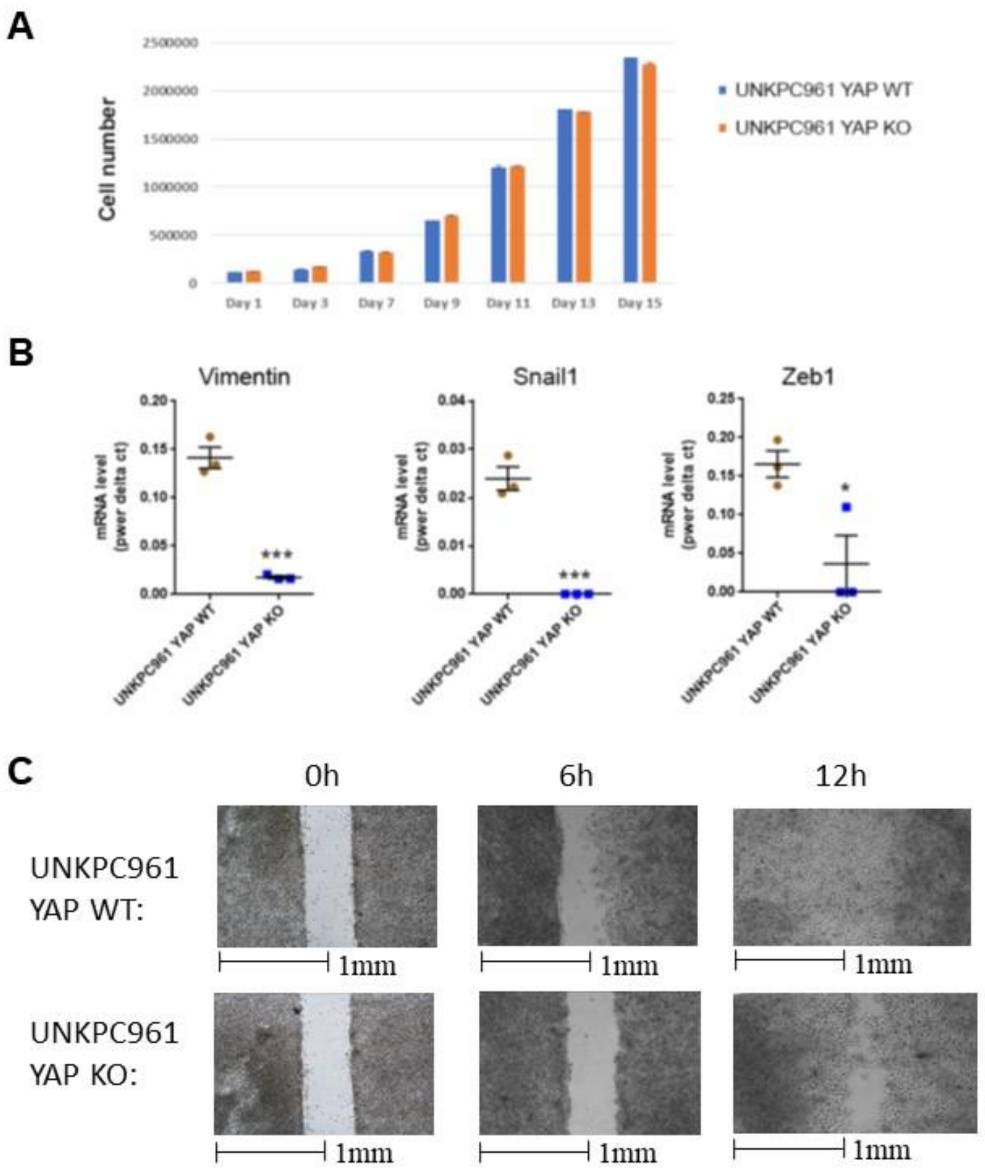

<u>Suppl Fig 7.</u> YAP KO decreases PDAC cell EMT and migration.
Number of UNKPC961 YAP WT and KO cells for up to 15 days (A). mRNA levels of EMT markers in UN-KPC961-Luc wild type and YAP KO PDAC cells (B). Migration assay of UN-KPC961-Luc wild type and YAP KO PDAC cells (C). *, $p < 0.05$ *versus* YAP WT. ***, $p < 0.005$ *versus* YAP WT.

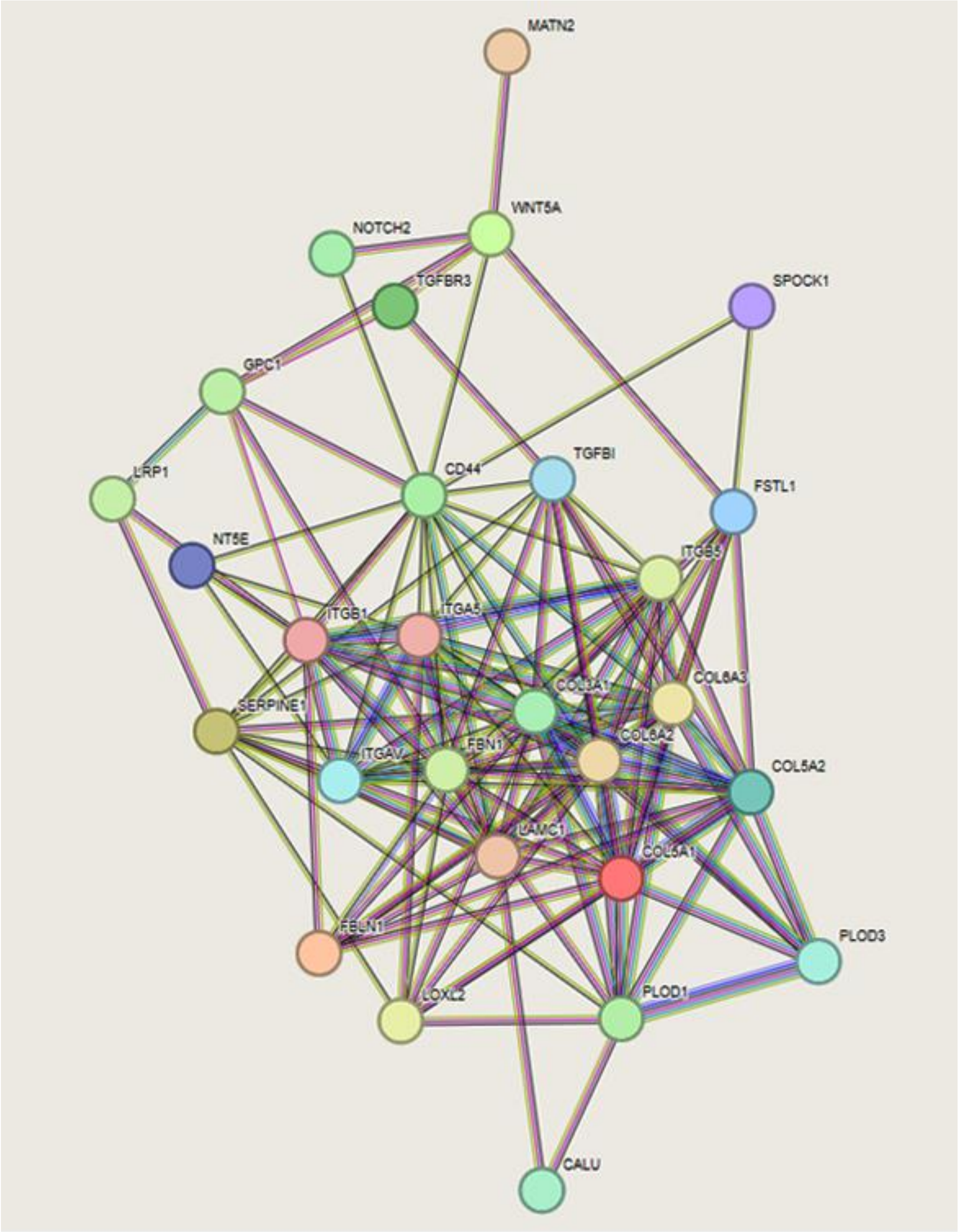

<u>Suppl Fig 8.</u> Genes set enrichment map of RNAseq data using the pathway-to-pathway network expression map.
